# PKAc-directed interaction and phosphorylation of Ptc is required for Hh signaling inhibition in *Drosophila*

**DOI:** 10.1038/s41421-019-0112-z

**Published:** 2019-09-10

**Authors:** Jialin Fan, Yajie Gao, Yi Lu, Wenqing Wu, Shuo Yuan, Hailong Wu, Dahua Chen, Yun Zhao

**Affiliations:** 1 State Key Laboratory of Cell Biology, CAS Center for Excellence in Molecular Cell Science, Innovation Center for Cell Signaling Network, Shanghai Institute of Biochemistry and Cell Biology, Chinese Academy of Sciences, University of Chinese Academy of Sciences, Shanghai, China; 2 State Key Laboratory of Membrane Biology, Institute of Zoology, Chinese Academy of Sciences, Beijing, China; 3 School of Life Science and Technology, ShanghaiTech University, Shanghai, China

**Keywords:** Growth factor signalling, Developmental biology, Growth factor signalling, Developmental biology

## Abstract

Ptc is a gatekeeper to avoid abnormal Hh signaling activation, but the key regulators involved in Ptc-mediated inhibition remain largely unknown. Here, we identify PKAc as a key regulator required for Ptc inhibitory function. In the absence of Hh, PKAc physically interacts with Ptc and phosphorylates Ptc at Ser-1150 and -1183 residues. The presence of Hh unleashes PKAc from Ptc and activates Hh signaling. By combining both in vitro and in vivo functional assays, we demonstrate that such Ptc–PKAc interaction and Ptc phosphorylation are both important for Ptc inhibitory function. Interestingly, we further demonstrate that PKAc is subjected to palmitoylation, contributing to its kinase activity on plasma membrane. Based on those novel findings, we establish a working model on Ptc inhibitory function: In the absence of Hh, PKAc interacts with and phosphorylates Ptc to ensure its inhibitory function; and Hh presence releases PKAc from Ptc, resulting in Hh signaling activation.

## Introduction

As one of the most evolutionarily conserved morphogen pathways, the Hedgehog (Hh) signaling pathway plays vital roles in embryo development, tissue regeneration, and homeostasis^1,2^. Aberrant inactivation or activation of Hh signaling is associated with various human congenital disorders and cancers^3,4^. One of the key features which distinguishes Hh signaling with other signaling pathways is the existence of an important inhibitory receptor, a 12-transmembrane protein Patched (Ptc). Physiologically, in the absence of ligands, Ptc inhibits the activation of a downstream seven-transmembrane signal transducer, Smoothened (Smo), and consequently silences the Hh signaling^5^. Conversely, in the presence of Hh, Ptc binds to Hh and releases Smo from inhibition, resulting in the activation of Hh signaling as evidenced by accumulation of Ci full-length and induction of downstream target genes of Hh signaling including *dpp*, *ptc*, and *en*^5^. Therefore, the inhibitory function of Ptc is critical in regulating the switch-off and -on of Hh signaling and is inevitably subjected to precise regulation at multiple levels to guarantee the appropriate Hh signaling activity in Hh-responsive cells.

Previous studies have demonstrated that Hh signaling is abnormally activated in many types of cancers including basal cell carcinomas (BCCs), medulloblastomas (MBs) and non-small cell lung carcinomas^6–8^. In those Hh-driven malignancies, loss of the inhibitory function of Ptc is the predominant alteration leading to abnormal Hh activation. For example, over 70% sporadic BCCs possess Ptc mutations^9^; and mutations inactivating Ptc inhibitory function have been reported in a subset of sporadic MBs^10^. Moreover, although the loss of both copies of *Ptc* is embryonic lethal, one copy loss of *Ptc* is sufficient to induce MBs in mouse models^11^. Therefore, those findings from both clinical samples and animal models strongly indicate the physiological and pathological importance of Ptc-mediated inhibition on Hh signaling. A long-standing idea believes that Ptc inhibits Hh signaling in a catalytic manner by pumping activating sterols away from Smo^12^, but whether alterative mechanisms are involved in Ptc inhibitory function remains elusive.

Post-translational modifications (PTMs) play a key role in intracellular signal transduction. Like other signaling pathways, including Wnt, Notch, and Hippo^13–15^, Hh signaling is extensively subjected to multiple PTMs^16^. Among them, phosphorylation is one of most important PTMs in regulating Hh signaling activity. When Hh signaling is off, cyclic adenosine monophosphate (cAMP)-dependent protein kinase A (PKA) phosphorylates Ci and primes it for the subsequent phosphorylation by glycogen synthase kinase 3 (GSK3) and casein kinase 1 (CK1), resulting in phosphorylation-dependent ubiquitination and partial degradation of Ci full-length (Ci^fl^) to Ci repressor (Ci^R^)^17,18^. However, when Hh signaling is on, PKA phosphorylates Smo at its C terminus and triggers the subsequent adjacent phosphorylation of Smo by CK1 and GPCR kinase 2 (Gprk2) in *Drosophila*, which leads to plasma membrane accumulation and active conformation changes of Smo^19,20^. Therefore, PKA is one of the key regulators on Hh signaling activity.

PKA ubiquitously expresses in eukaryotic cells and functions as a signal switch in regulating the on and off of various signaling pathways, including Wnt, Hippo, and Hh^21,22^. The kinase activity of PKA is regulated by cAMP, an intracellular second messenger. In the absence of cAMP, PKA exists as an inactive tetramer composed of a regulatory (R) subunit dimer and two catalytic (C) subunits^23^. In the presence of cAMP, the two catalytic (C) subunits are released from their inactive tetramer and phosphorylate their specific substrates^23^. It is a long-standing concept that the subcellular distribution of PKA is largely determined by a family of structurally diverse proteins, so called A-kinase anchoring proteins (AKAPs), which confine PKA at specific cellular locations by binding to the regulatory (R) subunits of PKA^24,25^. However, some recent studies have indicated that the catalytic (C) subunits of PKA (PKAc) may have their distinct cellular distribution patterns independent on PKA regulatory (R) subunit dimer or AKAPs due to various posttranslational modifications including myristylation and deamidation^26–28^. For example, the myristylation at PKAc N terminus helps its plasma membrane localization^26,28^, and the deamidation at Asn2 supports PKAc to localize in the nucleus^27^. Given the importance of PKA in regulating Hh signaling and its potential for plasma membrane localization, we hypothesized that PKA might serve as a key regulator for the inhibitory function of Ptc.

In the current study, we demonstrate that, in the absence of Hh, PKAc can physically interact with Ptc at its 4th intracellular domain (C4) and phosphorylate the 7th intracellular domain of Ptc (C7). Interestingly, such a Ptc–PKAc interaction is mitigated in the presence of Hh ligands parallel with enhanced Smo-PKA interaction and the subsequent Smo activation. Following functional experiments indicate that both Ptc–PKAc interaction and PKAc-mediated Ptc phosphorylation are required for Ptc inhibitory function on Hh signaling. In addition, we also demonstrate that PKAc can be palmitoylated, and the palmitoylation is required for its plasma membrane localization and its physiological function on Ptc-mediated inhibition on Hh signaling. Therefore, we establish a working model for our newly identified Ptc–PKAc regulatory axis: in the absence of Hh, Ptc–PKAc interaction, and the subsequent PKAc-mediated Ptc phosphorylation are required for the inhibitory function of Ptc; in the presence of Hh, PKAc is released from Ptc, resulting in Hh signaling activation.

## Results

### Transmembrane receptor Ptc interacts with PKAc

**Fig. 1:**
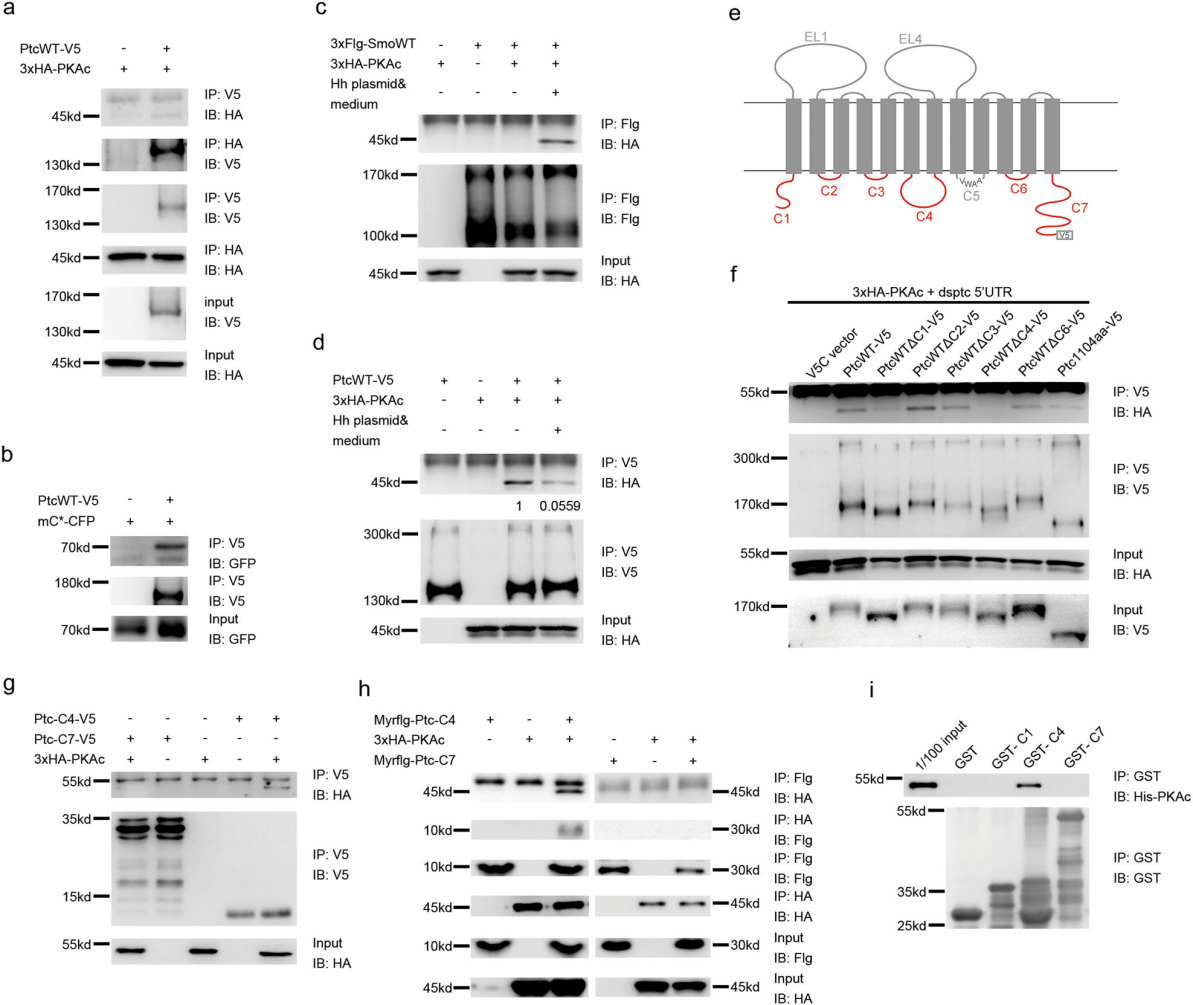
PKAc interacts with Ptc through Ptc’s intracellular domain C4, which could be regulated by Hh. Western blot analysis of immunoprecipitates or whole cell lysates from S2 cells expressing indicated constructs including PtcWT-V5 and 3XHA-PKAc (**a**) or mC*-CFP (**b**). S2 cells were transfected with the indicated constructs, treated with or without Hh medium, followed by co-Immunoprecipitation with anti-V5/Flg antibodies respectively. Western blot to detect interaction dynamics of SmoWT-PKAc (**c**) and PtcWT-PKAc (**d**). Hh-induced interaction reduction between Ptc–PKAc was quantified. **e** A simple cartoon of *Drosophila* transmembrane receptor PtcWT which marks its extracellular loops (EL1 and EL4) and intracellular domains (C1, C2, C3, C4, C5, C6, and C7). **f** S2 cells were transfected with dsRNA against *ptc* 5′UTR to knockdown endogenous Ptc and co-transfected with indicated expression constructs including 3XHA-PKAc, V5C vector, PtcWT-V5, PtcWTΔC1-V5, PtcWTΔC2-V5, PtcWTΔC3-V5, PtcWTΔC4-V5, PtcWTΔC6-V5, and PtcWTΔC7-V5. Immunoprecipitation assays to detect interaction between PKAc and indicated Ptc variants by western blot. **g** S2 cells were transfected with the indicated constructs including 3XHA-PKAc, Ptc-C4-V5, and Ptc-C7-V5, followed by co-Immunoprecipitation with anti-V5 antibodies. Western blot to detect interactions between PKAc and Ptc-C4 or Ptc-C7. **h** S2 cells were transfected with the indicated constructs including 3XHA-PKAc, membrane-tethered Ptc-C4 (Myrflg-Ptc-C4-V5, left half) and membrane-tethered Ptc-C7 (Myrflg-Ptc-C7-V5, right half), followed by co-Immunoprecipitation with anti-HA/Flag antibodies. Western blot to detect interactions between PKAc and Myrflg-Ptc-C4 or Myrflg-Ptc-C7. i GST pull-down assays were performed by using purified His-tagged PKAc (His-PKAc) and GST control or indicated GST-tagged Ptc intracellular domains (GST-C1, -C4, and -C7). Physical interactions of His-PKAc with those GST-tagged peptides were detected by western blot

Since PKA is deeply involved in Hh signaling transduction and shows the potential capacity of localizing at plasma membrane, we sought to test whether PKA functions as a novel regulator involved in Ptc-mediated Hh signaling inhibition. Due to the lack of commercial antibodies for *Drosophila* Ptc and PKAc, we have to examine the possible interaction between the PKAc and Ptc by overexpressing V5-tagged Ptc (PtcWT-V5) and HA tagged PKAc (3XHA-PKAc) in S2 cells followed with immunoprecipitation (IP) assays. Reciprocal IP assays by using capture antibodies against V5 or HA clearly showed co-IP between Ptc and PKAc (Fig. 1a). This Ptc–PKAc co-IP was further confirmed by IP assays between PtcWT-6xmyc and 3XHA-PKAc (Supplementary Fig. S1a). Given that a constitutively active mouse PKA catalytic subunit (mC*) is functional in *Drosophila* and *Drosophila* PKAc and mC* share more than 80% amino acid sequence identity^29^ (Supplementary Fig. S1b), we also performed IP assays to detect possible interaction between Ptc and mC*. As shown in Fig. 1b, Ptc–PKAc co-IP was confirmed between PtcWT-V5 and mC*-CFP. Consistent with previous studies^19^, we also found that PKAc can interact with Smo in the presence of Hh (Fig. 1c). We then started to examine whether the possible Ptc–PKAc interaction is regulated by the presence or absence of Hh. In contrast to the Smo-PKAc interaction which is enhanced in the presence of Hh (Fig. 1c), the Ptc–PKAc interaction was significantly diminished in the presence of Hh (S2 cells were simultaneously transfected with Hh plasmid and treated with Hh condition medium to maximize Hh stimulation) (Fig. 1d). These findings demonstrate the existence of a possible interaction between Ptc and PKAc, and indicate that such a Ptc–PKAc interaction is dynamically regulated in response to the absence or presence of Hh ligands.

### Ptc interacts with PKAc through its intracellular domain C4

As a 12-transmembrane domain protein, Ptc has seven intracellular domains, which we named them as C1–C7 from Ptc’s N terminus to C terminus hereafter (Fig. 1e). To further investigate which intracellular domains of Ptc contribute to its interaction with PKAc, we constructed a series of Ptc mutants with deletion of its intracellular domains (named PtcWT-ΔC1, -ΔC2, -ΔC3, -ΔC4, -ΔC6, and -ΔC7, respectively) (Fig. 1e). Because the Ptc-C5 (Fig. 1e, the gray loop at intracellular side) contains only four amino acid residues and is very unlikely responsible for the Ptc–PKAc interaction, we did not construct Ptc-ΔC5 deletion mutant (Fig. 1e). IP assays were then performed to detect interactions between those Ptc mutants and PKAc. During IP assays, endogenous Ptc was knocked down by using dsRNAs against Ptc 5′UTR ((Supplementary Fig. S1c) to avoid possible interactions of endogenous Ptc with those Ptc mutants which may mislead IP results^30^. The results from IP assays showed that although deletion of C1 (Fig. 1f, lane 3) or C7 (Fig. 1f, lane 8) reduced the Ptc–PKAc interaction, suggesting that C1 and C7 may facilitate Ptc–PKAc interaction, the Ptc intracellular domain C4 is essential for the Ptc–PKAc interaction because C4 deletion completely abolished the interaction of Ptc with PKAc (Fig. 1f, lane 6 and Supplementary Fig. S1d). In line with this notion, the intracellular domain C4 (Ptc-C4, from a.a 583 to a.a 670) but not the intracellular domain C7 (Ptc-C7, from a.a 1104 to a.a 1286) showed interaction with PKAc (Fig. 1g and Supplementary Fig. S1e). Since Ptc is a plasma membrane protein, we then started to examine interactions of PKAc with membrane-tethered Ptc-C4 (myrflg-Ptc-C4) and Ptc-C7 (myrflg-Ptc-C7). IP assays showed that only the myrflg-Ptc-C4 (Fig. 1h, left part) but not the myrflg-Ptc-C7 (Fig. 1h, right part) can interact with PKAc (Fig. 1h). More importantly, GST pull-down assays showed that only the domain C4 but not the domain C1 or C7 can directly interact with PKAc (Fig. 1i). Therefore, these findings indicate that Ptc directly interacts with PKAc and its intracellular domain C4 is essential for Ptc–PKAc interaction.

### The Ptc–PKAc interaction is required for Ptc inhibitory function on Hh signaling both in vitro and in vivo

**Fig. 2:**
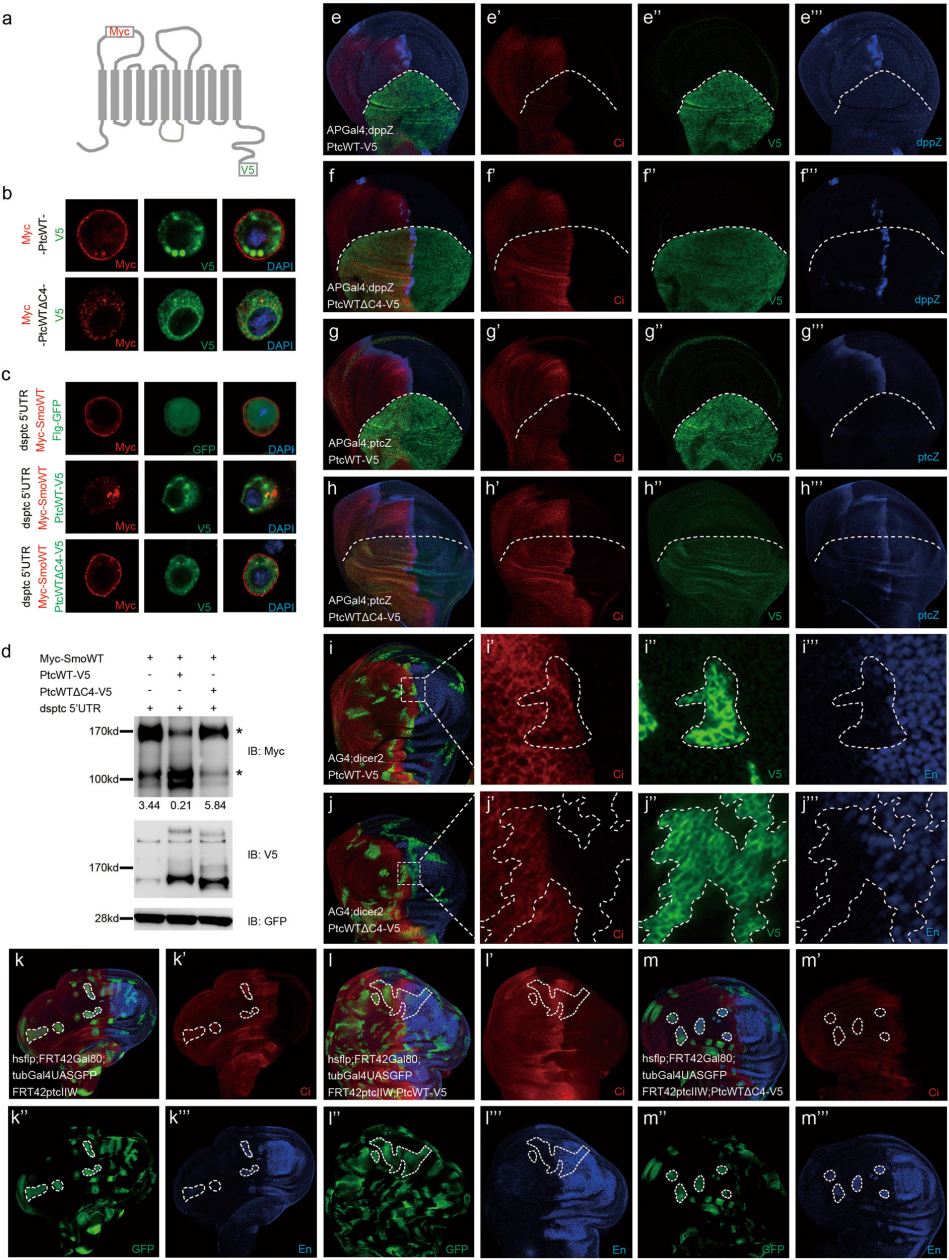
Intracellular domain C4 is required for Ptc inhibitory function on Hh signaling. **a** The cartoon model of Myc-PtcWT-V5 for staining, in which the Myc-tag was inserted into the extracellular linker 1 (EL1) region. **b** Immunofluorescent staining to detect the cellular distribution of Myc-PtcWT-V5 and Myc-PtcWTΔC4-V5 by staining Myc (red), V5 (green) tags, and DAPI (blue) in S2 cells without co-transfection of a PKAc plasmid. **c** Immunofluorescent staining to detect the cellular distribution of Myc-SmoWT (red) in the condition of enodgenous Ptc knockdown with dsRNA against ptc 5′UTR together with overexpressing Flg-GFP (GFP green), PtcWT-V5 (green), or PtcWTΔC4-V5 (green) in S2 cells without co-transfection of a PKAc plasmid. **d** S2 cells were transfected with dsRNA against ptc 5′UTR to knockdown endogenous Ptc and co-transfected with Myc-SmoWT together with PtcWT-V5 or PtcWTΔC4-V5. Myc-SmoWT dimerization was examined in S2 cells by western blot. The asterisks indicate the dimmer and monomer form of Myc-SmoWT respectively. Density ratio of Smo dimmer to monomer of each lane was indicated below. Immunostaining of wing discs expressing PtcWT-V5 (green) (**e**–**e‴**) or PtcWTΔC4-V5 (green) (**f**–**f‴**) driven by the dorsal compartment-specific driver AP-Gal4; Ci (red) and dpp-lacZ (dppZ) (blue) were immunostained to show the inhibition ability of PtcWT-V5 and PtcWTΔC4-V5 on Hh signaling in vivo. Immunostaining of wing discs expressing PtcWT-V5 (green) (**g**–**g”’**) or PtcWTΔC4-V5 (green) (**h**–**h‴**) driven by the dorsal compartment-specific driver AP-Gal4; Ci (red) and ptc-lacZ (blue) were immunostained to show the inhibition ability of PtcWT-V5 and PtcWTΔC4-V5 on Hh signaling in vivo. Immunostaining of clones in wing discs expressing PtcWT-V5 (green) (**i**–**i‴**) or PtcWTΔC4-V5 (green) (**j**–**j‴**) driven by the AG4; Ci (red) and En (blue) levels inside or outside of the clones were immunostained to show the inhibition ability of PtcWT-V5 and PtcWTΔC4-V5 on Hh signaling in vivo. Rescue assays to detect the rescue ability of PtcWT-V5 or PtcWTΔC4-V5 to loss-of-inhibition induced by endogenous Ptc depletion. Ci (red) and En (blue) levels were detected in GFP clones with endogenous Ptc depletion alone (**k**–**k‴**), together with PtcWT overexpression (**l**–**l‴**) or PtcWTΔC4 overexpression (**m**–**m‴**)

Given that Ptc–PKAc interaction was diminished in response to Hh (Fig. 1d), we started to examine whether Ptc–PKAc interaction contributes to Ptc inhibitory function on Hh signaling. As plasma membrane accumulation of Ptc is important for its inhibitory function, we firstly examined whether loss of Ptc–PKAc interaction could affect Ptc plasma membrane accumulation. PKAc overexpression greatly promoted the membrane accumulation of PtcWT but failed to increase that of PtcWTΔC4 (Supplementary Fig. S2a), suggesting that the Ptc–PKAc interaction may be essential for Ptc plasma membrane accumulation. To further confirm this notion, a Myc-tag was inserted into the extracellular loop 1 (EL1, Fig. 1e) of Ptc and a V5-tag was inserted into the C tail of Ptc, so that the plasma membrane localization of Ptc can be exclusively detected by anti-myc antibody through cell-surface staining (Fig. 2a), and the intracellular Ptc can be detected by anti-V5 antibody. Ptc-luciferase report assays showed that Myc-tag insertion did not affect Ptc inhibitory function on Hh signaling (Supplementary Fig. S2b). Cell-surface staining by an anti-Myc antibody was first performed and followed with intracellular V5 staining to show the cellular distribution of indicated Ptc proteins. In S2 cells overexpressing Myc-PtcWT-V5, the Myc signals were mainly detected at the plasma membrane and the V5 signals were apparently accumulated at the cytosol side surrounding the plasma membrane (Fig. 2b, upper half), suggesting the plasma membrane distribution of PtcWT. However, in S2 cells overexpressing C4-deleted Ptc (Myc-PtcWTΔC4-V5), both Myc and V5 signals showed diffuse intracellular patterns, suggesting the impaired plasma membrane localization of Myc-PtcWTΔC4-V5 (Fig. 2b, bottom half). These findings indicate the importance of Ptc–PKAc interaction on Ptc plasma membrane localization (Fig. 2b). Correspondingly, Ptc-luciferase assays showed the loss of the inhibitory function of PtcWTΔC4 on Hh signaling (Supplementary Fig. S2c). As Smo plasma membrane accumulation and dimerization are key features of Hh signaling activation^31,32^, we then examined whether Ptc–PKAc interaction loss could affect those features. IF staining showed that compared with the control (overexpression of flg-GFP and dsRNA of 5′UTR), overexpression of PtcWT can markedly decreased the plasma membrane accumulation of Smo, while PtcWTΔC4 cannot (Fig. 2c). Meanwhile, western blot assays showed that compared with PtcWT whose overexpression significantly inhibited Smo dimerization (Fig. 2d, lane 2), PtcWTΔC4 failed to inhibit Smo dimerization (Fig. 2d, lane 3). These findings collectively suggest that the Ptc–PKAc interaction is required for the inhibitory function of Ptc in vitro.

To further investigate whether the Ptc–PKAc interaction is critical for Ptc inhibitory function in vivo, we generated transgenic flies overexpressing either PtcWT or PtcWTΔC4 respectively, and examined their functions on Hh signaling activities. Compared with control flies, PtcWT overexpression driven by either APGal4 or AG4 greatly inhibited *dpp* (Fig. 2e–e**‴**, dpp-lacZ), *ptc* (Fig. 2g–g**‴**, ptc-lacZ), and En expression levels (Fig. 2i–i**‴**), whereas PtcWTΔC4 overexpression showed impaired inhibition on dpp-lacZ (Fig. 2f–f**‴**), ptc-lacZ (Fig. 2h–h**‴**) and En expression levels (Fig. 2j–j**‴**). Interestingly, in addition to the inability of PtcWTΔC4 to inhibit Hh signaling, PtcWTΔC4 overexpression induced *dpp* expression (Fig. 2f–f**‴**), suggesting the dominant negative role of PtcWTΔC4 and the C4 is important for Ptc inhibitory function. Moreover, loss of inhibition on Hh signaling was also observed in another line of PtcWTΔC4 transgenic flies driven by MS1096 or APGal4 (Supplementary Fig. S2d–i**‴**). More importantly, in vivo rescue assays showed that only the PtcWT (Fig. 2l–l**‴** and Supplementary Fig. S2j–j**‴**) but not the PtcWTΔC4 (Fig. 2m–m**‴** and Supplementary Fig. S2k–k**‴**) could rescue the inhibition loss on En expression caused by endogenous Ptc depletion (Fig. 2k–k**‴**). These findings suggest that Ptc–PKAc interaction is critical for Ptc inhibitory function on Hh signaling in vivo.

### PKAc-mediated phosphorylation on the Ptc C-tail (domain C7) is important for Ptc inhibitory function on Hh signaling in vitro and in vivo

As a well characterized protein kinase, PKA can phosphorylate numerous substrates including Smo and Ci in response to diverse external and internal signals. Although PKA normally phosphorylates its target proteins at consensus PKA sites, PKA phosphorylation at the nonconsensus PKA sites has been reported previously^33^. Since we have demonstrated the physical interaction between Ptc and PKAc, we sought to determine whether Ptc is a novel phosphorylation substrate of PKAc. Compared with the vehicle control (Supplementary Fig. S3a, lane 1 of both left and right half), okadaic acid treatment retarded migration of either Myc- (left half) or V5-tagged Ptc (right half) proteins (Supplementary Fig. S3a, lane 2 of both and right half), which could be reversed by treatment of phosphatases, such as FastAP or Cip (Supplementary Fig. S3a, lane 3 and 4 of both left and right half). This finding suggests that Ptc is subjected to phosphorylation modification.

**Fig. 3:**
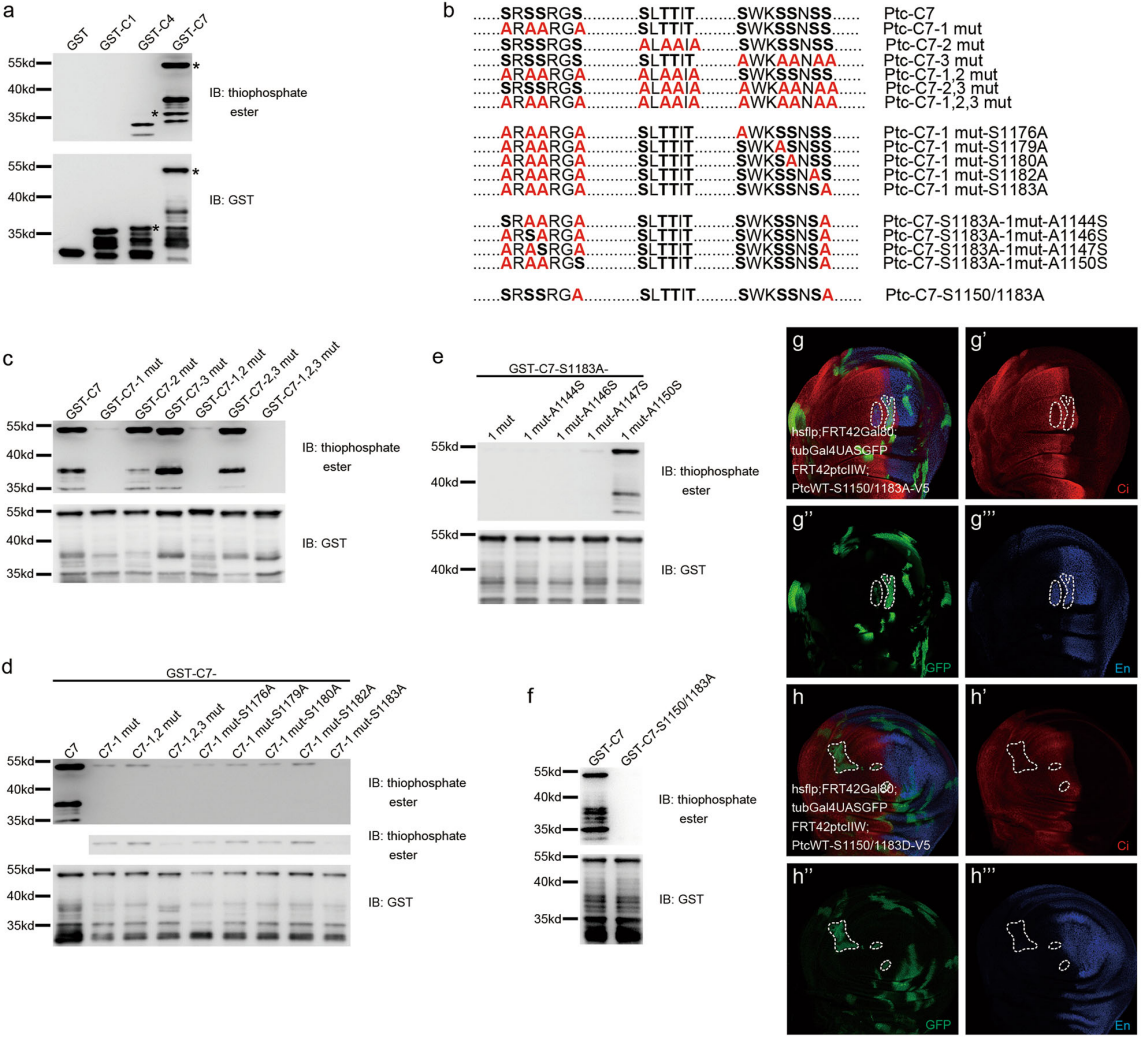
PKAc can phosphorylate Ptc at its C terminal domain. **a** In vitro ATPγS phosphorylation assays for purified GST-Ptc-C1, GST-Ptc-C4, and GST-Ptc-C7. The asterisks marked the GST-ptc-C4 or GST-ptc-C7 bands. **b** The amino acid sequence information of putative phosphorylation sites on Ptc-C7 detected by mass spectrometry analysis and the annotation of indicated Ptc mutants. In vitro ATPγS phosphorylation assays to define phosphorylable clusters (**c**), phosphorylation sites at phospho-cluster 3 (**d**), and 1 (**e**). Validation of the key phosphorylation sites (Ser-1150 and -1183) at the Ptc C terminus (**f**). Rescue assays to detect the rescue ability of PtcWT-V5 or PtcWTΔC4-V5 to loss-of-inhibition induced by endogenous Ptc depletion. Ci (red) and En (blue) levels were detected in GFP clones (green) in the condition of endogenous Ptc depletion together with overexpression of either PtcWT-S1150/1183A-V5 (**g**–**g‴**) or PtcWT-S1150/1183D-V5 (**h**–**h‴**)

To further examine whether PKAc can phosphorylate Ptc, we performed in vitro kinase assays (thiophosphorylation) by PKAc in the presence of purified Ptc-C1, -C4 and -C7 intracellular domains (Fig. 1e), which are most likely subjected to phosphorylation modifications (Fig. 3a). Thiophosphorylation assays showed that PKAc can phosphorylate Ptc at both C4 and C7 domains (Fig. 3a, upper half, lane 3 and 4), but the thiophosphorylation band at C4 was not corresponding to GST-C4 full length (Fig. 3a, marked by asterisks). In addition, follow-up mass spectrometry (MS) analysis only detected phosphorylation sites at domain C7 but not C4 (Supplementary Fig. S3b). All these findings suggest that the thiophosphate ester signals on domain C4 may be false positive (Fig. 3a, upper half, lane 3). After sequence analysis of the phosphorylation sites on domain C7, we grouped them into three phospho-clusters (Fig. 3b). To examine which phospho-cluster(s) contribute(s) to PKAc-mediated Ptc phosphorylation, we introduced mutations to substitute phosphorylable Ser/Thr to unphosphorylable Ala at single, both or all three phospho-clusters (Fig. 3b). Thiophosphorylation assays by PKAc showed that substitution mutations at phospho-cluster 2 (GST-C7-2 mut) (Fig. 3c, lane 3), phospho-cluster 3 (GST-C7-3 mut) (Fig. 3c, lane 4) or both 2 and 3 (GST-C7-2,3 mut) (Fig. 3c, lane 6) did not affect the phosphorylation levels of Ptc, but substitution mutations at phospho-cluster 1 (GST-C7-1 mut) (Fig. 3c, lane 2) or both 1 and 2 phospho-clusters (GST-C7-1,2 mut) (Fig. 3c, lane 5) led to a dramatic phosphorylation decrease in Ptc protein (Fig. 3c). Moreover, substitution mutations at all three phospho-clusters (GST-C7-1,2,3 mut) completely blocked PKAc-mediated Ptc phosphorylation (Fig. 3c, lane 7). These results suggested that the phospho-cluster 1 and 3 may contain PKAc-phosphorylation sites.

To precisely determine the PKAc-phosphorylation sites on Ptc, we separately substituted all five Ser residues in phospho-cluster 3 with Ala in the backbone of ‘GST-C7-1 mut’, in which all Ser residues in phospho-cluster 1 are mutated to Ala (Fig. 3b). Thiophosphorylation assays showed that compared with other four substitution mutations at Ser-1176, -1179, -1180, and -1182 (Fig. 3d, lane 5–8), the substitution mutation at Ser-1183 completely abolished PKAc-mediated phosphorylation at the GST-C7-1 mut background (Fig. 3d, lane 9), suggesting that the Ser-1183 is the PKAc-phosphorylation site at the phospho-cluster 3 of Ptc-C7. Since PKAc was not able to phosphorylate the fragment GST-C7-1 mut-S1183A (with substitution mutations at all Ser residues of phospho-cluster 1 and at Ser-1183 in the phospho-cluster 3) (Fig. 3d, lane 9), we then adopted this fragment to define the PKAc-phosphorylation sites at phospho-cluster 1 by back mutation from Ala residue to Ser residue. Compared with back mutations at Ala-1144, -1146, and -1147 (Fig. 3e, lane 2, 3, and 4), the back mutation at Ala-1150 restored the PKAc-mediated phosphorylation, suggesting that Ser-1150 is the PKAc-phosphorylation site at phospho-cluster 1 (Fig. 3e, lane 5). Since we determined that PKAc can phosphorylate Ptc at Ser-1150 and -1183 residues, we then constructed a Pct-C7 mutant (GST-C7-S1150/1183A) with substitution mutations at both Ser residues (Fig. 3b, the bottom fragment). Thiophosphorylation assays showed that PKAc failed to phosphorylate this mutant (Fig. 3f), further supporting that PKAc phosphorylates Ptc at the Ser-1150 and -1183 of the Ptc-C7 domain. Moreover, PKAc-mediated phosphorylation of Ptc at Ser-1150 and -1183 was further confirmed by MS assays, although the phosphorylation signals at Ser-1150 is moderate (Supplementary Fig. S3b).

In the absence of Hh, the plasma membrane accumulation of Ptc is very important for its inhibitory function. To examine whether PKAc-mediated Ptc phosphorylation is important for Ptc inhibitory function, we first performed immunofluorescent staining to compare the plasma membrane accumulation of unphosphorylable Ptc substitution mutant (PtcWT-S1150/1183A) and Ptc phosphomimetics (PtcWT-S1150/1183D). As shown in Supplementary Fig. S3c, the lack of PKAc-mediated phosphorylation greatly impaired Ptc plasma membrane localization. More importantly, our in vivo rescue assays showed that PtcWT-S1150/1183A failed to rescue the inhibition loss on En which is caused by endogenous Ptc depletion (Fig. 2k–k**‴**, Fig. 3g–g**‴** and Supplementary Fig. S3d–d**‴**), whereas PtcWT-S1150/1183D restored the inhibition on En (Fig. 2k–k**‴**, Fig. 3h–h**‴** and Supplementary Fig. S3e–e**‴**). Given that the Ptc–PKAc interaction was decreased in response to Hh ligands, we then examined whether PKAc-mediated Ptc phosphorylation is also regulated by Hh. MS data indicated that Hh treatment eliminate the phospho signals at Ser-1150 and greatly diminished them at Ser-1183 (Supplementary Fig. S3f). These findings indicate that PKAc can phosphorylate Ptc C-tail (domain C7) at Ser-1150 and -1183 and such PKAc-mediated Ptc phosphorylation is important for Ptc inhibitory function and regulated by Hh.

### Ptc–PKAc interaction and PKAc-mediated Ptc phosphorylation are both important for Ptc inhibitory function

**Fig. 4:**
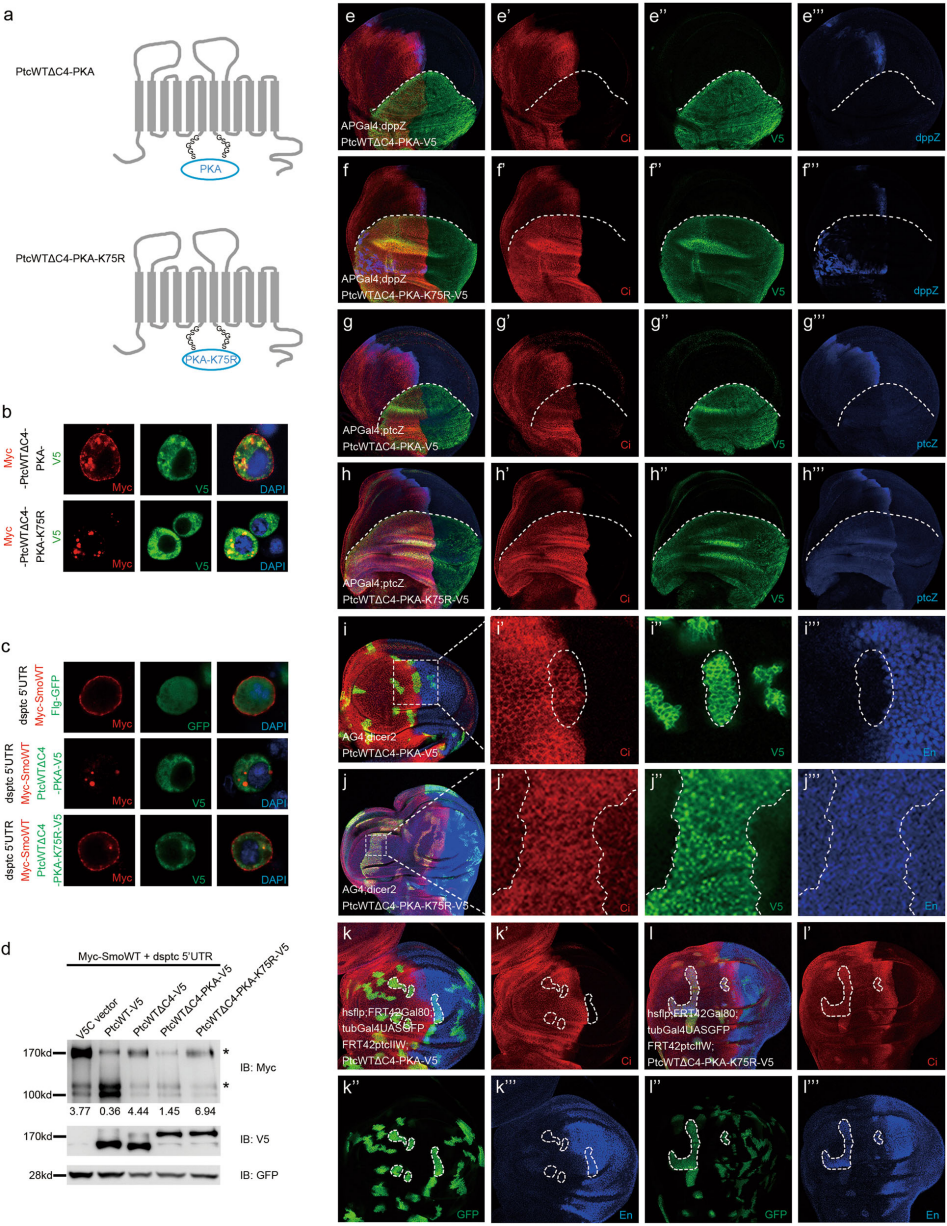
PKAc can replace C4 domain to maintain Ptc inhibition function in Hh pathway. **a** A simple cartoon of fusion Ptc proteins whose C4 domain was replaced with either PKAc (PtcWTΔC4-PKA-V5) or kinase-dead PKAc (PtcWTΔC4-PKA-K75R-V5). **b** Immunofluorescent staining to detect the cellular distribution Myc-PtcWTΔC4-PKA-V5 and Myc-PtcWTΔC4-PKA-K75R-V5 by staining Myc (red), V5 (green) tags, and DAPI (blue) in S2 cells without co-transfection of a PKAc plasmid. **c** Immunofluorescent staining to detect the cellular distribution of Myc-SmoWT (red) in the condition of endogenous Ptc knockdown with dsRNA against ptc 5′UTR together with overexpressing Flg-GFP (GFP green), PtcWT-V5 (green) or PtcWTΔC4-V5 (green) in S2 cells without co-transfection of a PKAc plasmid. d S2 cells were transfected with dsRNA against ptc 5′UTR to knockdown endogenous Ptc and co-transfected with Myc-SmoWT together with PtcWT-V5, PtcWTΔC4-V5, PtcWTΔC4-PKA-V5, and PtcWTΔC4-PKA-K75R. Myc-SmoWT dimerization was examined in S2 cells by western blot. The asterisks indicate the dimmer and monomer form of Myc-SmoWT respectively. Density ratio of Smo dimmer to monomer of each lane was indicated below. Immunostaining of wing discs expressing PtcWTΔC4-PKA-V5 (green) (**e**–**e‴**) or PtcWTΔC4-PKA-K75R-V5 (green) (**f**–**f‴**) driven by the dorsal compartment-specific driver AP-Gal4; Ci (red) and dpp-lacZ (blue) were immunostained to show the inhibitory effects of PtcWTΔC4-PKA-V5 or PtcWTΔC4-PKA-K75R-V5 on Hh signaling in vivo. Immunostaining of wing discs expressing PtcWTΔC4-PKA-V5 (green) (**g**–**g‴**) or PtcWTΔC4-PKA-K75R-V5 (green) (h-h”’) driven by the dorsal compartment-specific driver AP-Gal4; Ci (red) and ptc-lacZ (blue) were immunostained to show the inhibitory effects of PtcWTΔC4-PKA-V5 or PtcWTΔC4-PKA-K75R-V5 on Hh signaling in vivo. Immunostaining of clones in wing discs expressing PtcWTΔC4-PKA-V5 (green) (**i**–**i‴**) or PtcWTΔC4-PKA-K75R-V5 (green) (**j**–**j‴**) driven by the AG4; Ci (red) and En (blue) levels inside or outside of the clones were immunostained to show the inhibition ability of PtcWTΔC4-PKA-V5 and PtcWTΔC4-PKA-K75R-V5 on Hh signaling in vivo. Rescue assays to detect the rescue ability of PtcWTΔC4-PKA-V5 or PtcWTΔC4-PKA-K75R-V5 to loss-of-inhibition induced by endogenous Ptc depletion. Ci (red) and En (blue) levels were detected in GFP clones with Ptc depletion together with PtcWTΔC4-PKA-V5 overexpression (**k**–**k‴**) or PtcWTΔC4-PKA-K75R-V5 overexpression (**l**–**l‴**)

To further confirm our findings that both Ptc–PKAc interaction and PKAc-mediated Ptc phosphorylation are important for Ptc inhibitory function, we replaced the Ptc domain C4 with wild-type PKAc (PtcWTΔC4-PKA) or its kinase dead form, PKAc-K75R (PtcWTΔC4-PKA-K75R, in which the 75th residue Lysine, K, in PKAc active center was mutated to residue Arginine, R) to mimic Ptc interacting with PKAc or kinase-dead PKAc. A ‘soft linker (GSGGS)’ was included between Ptc and PKAc to avoid possible structure alterations in the Ptc–PKAc fusion protein (Fig. 4a). In addition, a Myc-tag and a V5-tag were inserted into the Ptc–PKAc fusion proteins using the same strategy in Fig. 2a. Unlike PtcWTΔC4 who has impaired plasma membrane localization ability (Fig. 2b, bottom half), IF staining showed that PtcWTΔC4-PKA has normal plasma membrane distribution as PtcWT (Fig. 4b, upper half), whereas C4 replacement with kinase-dead PKAc (PtcWTΔC4-PKA-K75R) resulted in significantly impaired plasma membrane localization of Ptc (Fig. 4b, bottom half), further supporting that Ptc–PKAc interaction and PKAc-mediated Ptc phosphorylation are important for Ptc plasma membrane localization. At the same time, compared with the flg-GFP control (Fig. 4c, upper part), overexpression of PtcWTΔC4-PKA (Fig. 4c, middle part) but not PtcWTΔC4-PKA-K75R (Fig. 4c, bottom part) blocked Smo plasma membrane accumulation. We found that PtcWTΔC4-PKA (Fig. 4d, lane 4) but not PtcWTΔC4-PKA-K75R (Fig. 4d, lane 5) could inhibit Smo dimerization in S2 cells, although PtcWTΔC4-PKA showed less effective than PtcWT in inhibiting Smo dimerization. Ptc-luciferase assays showed that Ptc proteins with either C4-deletion (PtcWTΔC4), C4-replacement by PKAc-K75R (PtcWTΔC4-PKA-K75R) or unphosphorylable mutation (PtcWT-S1150/1183A) were unable to inhibit Hh-induced signaling activation, whereas Ptc proteins with either C4-replacement by PKAc (PtcWTΔC4-PKA) or phosphomimetics (PtcWT-S1150/1183D) displayed inhibition ability (Supplementary Fig. S4a). More importantly, in vivo IF staining of wing discs from transgenic flies demonstrated that compared with PtcWTΔC4-PKA that inhibit *dpp* (Fig. 4e–e**‴** and Supplementary Fig. S4b–b**‴**, dpp-lacZ), *ptc* (Fig. 4g–g**‴** and Supplementary Fig. S4d–d**‴**, ptc-lacZ) and En (Fig. 4i–i**‴** and Supplementary Fig. S4f–f**‴**) expression, PtcWTΔC4-PKA-K75R functions as a dominant negative Ptc mutant to induce the expression of *dpp* (Fig. 4f–f**‴** and Supplementary Fig. S4c–c**‴**, dpp-lacZ), *ptc* (Fig. 4h–h**‴** and Supplementary Fig. S4e–e**‴**, ptc-lacZ) and En (Fig. 4j–j**‴** and Supplementary Fig. S4g–g**‴**) expression levels. In addition, compared with control fly (Supplementary Fig. S5a–a′, S5b–b′), phosphomimetics (PtcWT-S1150/1183D) but not unphosphorylable Ptc mutant (PtcWT-S1150/1183A) showed inhibition on Hh signaling as evidenced by reduced levels of *dpp* (Supplementary Fig. S5c–c**‴** vs. S5d–d**‴**) and ptc (Supplementary Fig. S5e–e**‴** vs. S5f-f**‴**) expression levels. Meanwhile, rescue assays showed that PtcWTΔC4-PKA (Fig. 4k–k**‴** and Supplementary Fig. S4h–h**‴**) but not PtcWTΔC4-PKA-K75R (Fig. 4l–l**‴** and Supplementary Fig. S4i–i**‴**) could restore Ptc-mediated inhibition on Hh signaling. Therefore, these findings indicate that both Ptc–PKAc interaction and PKAc-mediated Ptc phosphorylation are important for Ptc-mediated inhibition on Hh signaling.

### Palmitoylated PKAc contributes to its plasma membrane location and spatial kinase activity on Ptc and Smo

**Fig. 5:**
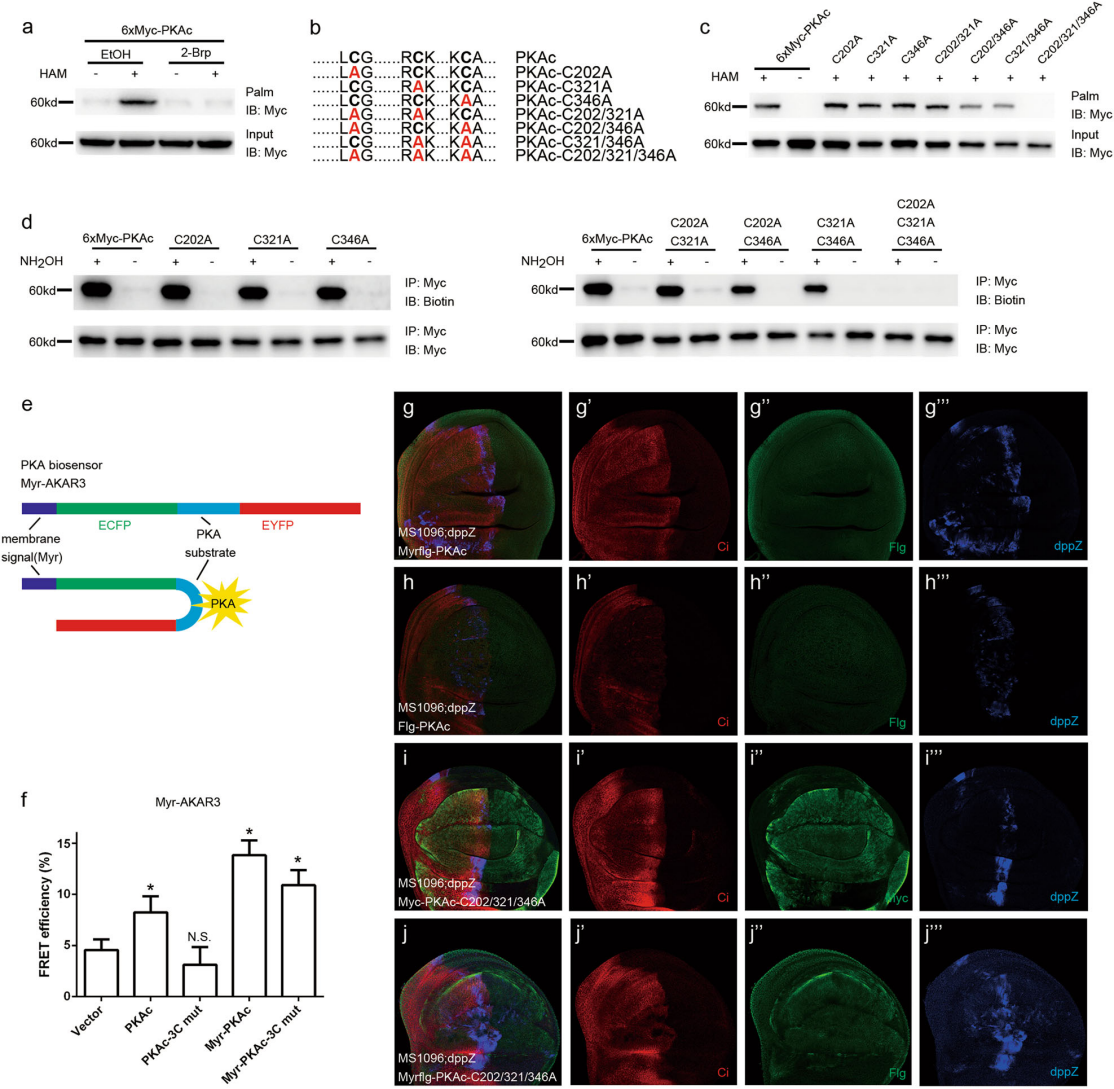
Palmitoylated PKAc can locate on membrane and upregulate Hh signaling. **a** S2 cells were transfected with the 6XMyc-PKAc expression construct. After 42 h of transfection, cells were treated with ethyl alcohol (as a control) or 2-Brp (25 μM) for 6 h; cell lysates were immunoprecipitated with anti-Myc beads, and then subjected to the S-palmitoylation assay to measure palmitoylation levels of PKAc. HAM and Palm are abbreviations for hydroxylamine and palmitoylation, respectively. **b** Amino acid sequence information of predicted palmitoylation sites on PKAc and the annotation of a series of PKAc mutants. **c** S2 cells were transfected with 6XMyc-PKAc and indicated PKAc mutants to detect S-palmitoylation sites. **d** S2 cells were transfected with the 6XMyc-PKAc and indicated PKAc mutants including single site mutants (left half) and double- or triple-site mutants (right half). Cell lysates were immunoprecipitated with anti-Myc beads, and then subjected to the biotin switch assay to measure palmitoylation levels of PKAc. **e** The cartoon of Myr-AKAR3 FRET, Myr was short for myristoylation. **f** FRET efficiency from Myr-AKAR3 in S2 cells expressing soluble PKAc, soluble PKAc-C202/321/346A (or PKAc-3C mut), Myr-PKAc or Myr-PKAc-C202/321/346A (or Myr-PKAc-3C mut). Immunostaining of wing discs expressing Myrflg-PKAc (membrane-tethered PKAc, green, **g**–**g‴**), Flg-PKAc (soluble PKAc, green, **h**–**h‴**), Myc-PKAc-C202/321/346A (soluble palmitoylation null PKAc, green, **i**–**i‴**) or Myrflg-PKAc-C202/321/346A (membrane-tethered palmitoylation null PKAc, green, **j**–**j‴**) driven by the MS1096; Ci (red) and dpp-lacZ (blue) were immunostained to show the effects of the indicated PKAc and their mutants on Hh signaling in vivo

The plasma membrane localization of Ptc is important for its inhibitory on Hh signaling in the absence of Hh. As we have demonstrated that both Ptc–PKAc interaction and PKAc-mediated Ptc phosphorylation are important for Ptc inhibitory function, we hypothesized that such a Ptc–PKAc interaction should occur on plasma membrane. Although some previous studies have suggested that PKAc could localize at cell membrane^26,28^, the mechanism leading to PKAc membrane localization is still not fully understood. Given that palmitoylation is one of the important PTMs to enhance hydrophobicity and the subsequent membrane localization of many target proteins^34,35^, we started to examine whether PKAc can be palmitoylated. Thiopropyl captivation of S-palmitoylated protein assays showed that treatment of hydroxylamine but not control Tris–HCl greatly increased the association of purified PKAc with thiopropyl beads and such association was markedly reduced in the presence of 2-Brp, a palmitoylation inhibitor (Fig. 5a), suggesting that PKAc is subjected to palmitoylation. Sequence analysis of PKAc amino acids disclosed that PKAc contains 3 putative palmitoylation sites, PKAc-Cys202, -Cys321, and -Cys346 (Fig. 5b). Substitution mutations at each putative palmitoylation sites (from Cys to Ala: PKAc-C202A, -C321A, and -C346A) showed marginal effects on PKAc palmitoylation but substitution mutations of all those three palmitoylation sites (PKAc-C202/321/346A) completely abolished the palmitoylation of PKAc (Fig. 5c), suggesting that those three Cys residues on PKAc protein are redundant for palmitoylation. Moreover, IP-ABE assays further demonstrated that PKAc could be redundantly palmitoylated at Cys202, Cys321, and Cys346 residues (Fig. 5d). To determine whether PKAc palmitoylation contributes to its plasma membrane accumulation, we employed the membrane-tethered biosensor AKAR3 (A-kinase activity reporter 3), which can be phosphorylated by PKAc, for the Fluorescence Resonance Energy Transfer (FRET) assay^36^ (Fig. 5e). PKAc increased the AKAR3 FRET, whereas the palmitoylation null PKAc mutant (PKAc-3C mut) failed to do so (Fig. 5f). More importantly, either membrane-tethered PKAc or PKAc-3C mut greatly enhanced AKAR3 FRET (Fig. 5f). These findings suggest that palmitoylation of PKAc is important for PKAc membrane accumulation and the kinase activity of palmitoylation null PKAc mutant (PKAc-3C mut) remains intact.

To further determine whether PKAc palmitoylation affects Ptc inhibitory function, we determined the plasma membrane localization of both Ptc and Smo by IF staining assays. Compared with vector control, soluble or membrane-tethered PKAc greatly increased plasma membrane accumulation of Ptc and Smo, but PKAc-3C-mut failed to do so (Supplementary Fig. S6a–d). In the line with the finding that PKAc kinase activity is required for Ptc plasma membrane accumulation (Fig. 4b), treatment of H89, an inhibitor of PKAc, apparently reduced the Ptc plasma membrane accumulation (Supplementary Fig. S6a, c) but has limited effect on that of Smo (Supplementary Fig. S6b, d), suggesting that Ptc inhibitory function may largely rely on PKAc kinase activity, whereas Smo plasma membrane accumulation may be more resistant to PKAc kinase activity depletion. This resistance may result from the phosphorylation cascade on Smo which is subsequently mediated by PKAc, CK1, and Gprk2. In addition, the effect of PKAc palmitoylation on Hh signaling was examined in vivo. Consistent with previous studies showing that soluble PKAc can phosphorylate Ci to suppress Hh signaling^17^, overexpression of either soluble PKAc or palmitoylation null PKAc mutant (PKAc-3C mut) markedly inhibited Ci and dpp-lacZ expression, whereas overexpression of membrane-tethered PKAc or PKAc-3C mut has no effect on Ci levels but can greatly increase dpp-lacZ levels (Fig. 5g–j**‴**). These findings indicate that PKAc is subjected to palmitoylation and such palmitoylation is important for PKAc plasma membrane localization and plasma membrane activity of PKAc on Ptc.

According to these findings, we developed a working model on PKAc-mediated Ptc inhibitory function: (i) in the absence of Hh, PKAc interacts with and phosphorylates Ptc to ensure Ptc inhibitory function on Hh signaling and (ii) in the presence of Hh, PKAc is released from Ptc, resulting in activation of Hh signaling.

## Discussion

Hh signaling is one of the most important morphogen pathways in regulating embryo development and maintaining adult tissue homeostasis. Ptc-mediated inhibition on Hh signaling is a key step to ensure the proper Hh signaling activity because loss-of-function mutations of Ptc account for over 70% sporadic BCCs and part of medulloblastoma^9,10^. Unlike Ptc whose inhibitory function is mainly attributed to its inhibition on Smo, PKAc can differentially regulate Hh signaling activity by functionally and/or physically interacting with and phosphorylating multiple key components of Hh signaling including Ci and Smo. In contrast to PKAc-mediated Smo phosphorylation which activates Hh signaling in response to Hh, in present study we demonstrated that Ptc–PKAc interaction and PKAc-mediated Ptc phosphorylations at Ser-1150 and -1183 are both important for Ptc inhibitory function in the absence of Hh ligand (Fig. 3), but the mechanism by which those phosphorylations contribute to Ptc inhibitory function is unknown. One possibility is that those phosphorylations may stabilize Ptc by blocking its ubiquitin degradation by Smurf family E3 ubiquitin ligases^37^. Given that Hh-bound Ptc showed the reduced PKAc binding (Fig. 1d), it might be possible that Hh-bound Ptc may have reduced phosphorylation levels at Ser-1150 and -1183, facilitating the subsequent Ptc endocytosis and/or ubiquitination. In fact, eliminated phosphorylation at Ser-1150 and reduced phosphorylation at Ser-1183 were validated by MS assays (Supplementary Fig. S3b, f).

As Ptc is a 12-transmembrane receptor of the Hh signaling pathway and the plasma membrane accumulation is required for its inhibitory function, it is therefore necessary that PKAc-mediated regulation on Ptc should occur at plasma membrane. Previous studies have shown that the plasma membrane localization of PKAc can be achieved by membrane AKAPs or N-terminal myristoylation on PKAc subunits^14,24,26,28,36,38–40^. In present study, we further demonstrate that PKAc is also subjected to palmitoylation at Cys202, Cys321, and Cys346 and such modifications are important for PKAc plasma membrane localization and function as a key regulator of Ptc and Hh signaling. Amino acid sequence analysis indicated that the palmitoylatable Cys202 and Cys346 of *Drosophila* PKAc are conserved in its human analog, suggesting that human PKAc may be also subjected to palmitoylation for its plasma membrane localization and regulation on Ptc. Although both myristoylation and palmitoylation facilitate PKA plasma membrane localization, we could not rule out the possibility that myristoylation and palmitoylation may differentially affect PKA function, because palmitoylation null PKAc completely blocked PKAc plasma membrane localization (Fig. 5f). One interesting phenomenon in the present study is that both membrane-tethered and soluble PKAc can increase plasma membrane accumulation of Ptc and Smo (Supplementary Fig. S6a–d), which is very likely caused by excessive plasma membrane PKAc-mediated concurrent plasma membrane accumulation of Ptc and Smo. More interestingly, although both Ptc and Smo are accumulated at plasma membrane in response to the overexpression of either membrane-tethered PKAc or soluble PKAc, only membrane-tethered PKAc can activate Hh signaling and soluble PKAc shows inhibition on Hh signaling instead (Fig. 5g, h). Together with a previous study showing that the PKAc-unphosphorylable Smo mutant, SmoSA, fails to localize at plasma membrane and activate Hh signaling even in the presence of Hh^19^, these findings suggest the importance of membrane-associated PKAc in activating Hh signaling. This functional discrepancy of membrane-tethered PKAc and soluble PKAc can be explained by soluble PKAc-mediated process from Ci^fl^ to Ci^R^. In addition, the findings suggest a therapeutic potential of blocking PKAc palmitoylation in treatment of Hh-driven malignancies.

In the present study, we demonstrated that PKAc can phosphorylate Ser-1150 and Ser-1183 of Ptc, and this PKAc-mediated Ptc phosphorylation is important for Ptc inhibitory function because unphosphorylable Ptc (PtcWT-S1150/1183A) showed impaired plasma membrane accumulation in S2 cells (Supplementary Fig. S3c), and failed to execute its inhibitory function in vivo (Fig. 2k–k**‴**, Fig. 3g–g**‴** and Supplementary Fig. S3d–d**‴**). But the fact that the flank amino acid sequences of Ser-1150 and Ser-1183 are not consensus PKA sites raised the concern whether those Ptc phosphorylation is directly mediated by PKAc. To address this concern, we constructed fusion proteins by fusing Ptc with PKAc and kinase-dead PKAc (PKA-K75R) respectively. Like the unphosphorylable Ptc (PtcWT-S1150/1183A), Ptc fused with PKA-K75R showed impaired inhibitory function compared with the Ptc–PKAc fusion protein (Fig. 4 and Supplementary Figs. S4, 5). These results clearly showed that PKAc can directly phosphorylate Ptc and sustain Ptc’s inhibitory function.

In summary, in the present study we demonstrated that PKAc is required for the Ptc inhibitory function. In the absence of Hh, PKAc interacts with Ptc through its intracellular C4 domain and phosphorylated Ptc at its intracellular C7 domain at Ser-1150 and -1183 residues. Such a Ptc–PKAc interaction is reduced in the presence of Hh ligands parallel with enhanced Smo-PKAc interaction and Hh signaling activation. We further demonstrated that Ptc–PKAc interaction and PKAc-mediated Ptc phosphorylation are both required for Ptc plasma membrane localization and its inhibitory function on Hh signaling, because either PtcΔC4 or PtcΔC4-PKAc-K75R functions as dominant negative forms to impair PtcWT inhibitory function on Hh signaling. In addition, we demonstrated that PKAc can be palmitoylated at Cys-202, -321 and -346 residues. Palmitoylated PKAc contributes to plasma membrane accumulation of both Ptc and Smo and Hh signaling activation.

Given the key function of Ptc in regulating Hh signaling activity, lots of efforts have been invested to decipher the mechanism of Ptc’s inhibitory function, and several recent milestone studies have even disclosed the protein structures of Ptc^41–43^, which greatly promote the investigations on the mechanism study of Ptc inhibitory function. Some structure-based studies have proposed that Ptc may function as a sterol pump to remove sterol from binding with Smo resulting in inhibition on Smo activity^41,43,44^. Our proposed working model and the Ptc-pump model may work parallelly or in a crosstalk manner. If these two models crosstalk each other, it will be of great interests to explore whether PKAc-directed interaction and phosphorylation of Ptc somehow enhances the pump ability of Ptc is an interesting question worthy further investigation.

## Materials and methods

The GST fusion protein pull-down assay, S-Palmitoylation assay, biotin switch assay, in vitro phosphorylation kinase assay using ATP analog ATPγS, MS analysis of phosphorylation sites in vitro and in vivo, and transgenic flie generation were performed according to standard protocols. For the detailed procedures and fly stocks used in this study, see the Supplemental Information.

### Immunostaining for *Drosophila* wing discs

All the flies and larvae were cultured under standard fly culture conditions. First, cut third-instar larvae in 2/3 and fixed in freshly made 4% formaldehyde in PBS buffer at room temperature, rotated for 30 min. Second, washed the larvae for three times with buffer PBST (PBS, 0.1% Triton), 20 min each time. Third, blocked the larvae with 1%BSA in PBST for 15 min. Forth, incubated the larvae with primary antibody diluted in PBST for overnight at 4 °C. Fifth, washed with PBST three times again and incubated with secondary antibody diluted in PBST for 2 h at room temperature. Finally, washed the larvae for another three times, dissected wing discs and mounted in 40% glycerol. A Leica LAS SP8 confocal microscope was employed to take immunostaining images.

Primary antibodies used in this study: rabbit/mouse anti-V5 (Invitrogen), rabbit/mouse anti-Flag (Sigma), rabbit/mouse anti Myc (Sigma), rabbit/mouse HA (Sigma), rabbit/mouse anti lacZ (Sigma), rat anti-Ci (2A1, DSHB), mouse anti-Ptc (DSHB), mouse anti-Smo (DSHB), and mouse anti-En (DSHB).

### *Drosophila* cell culture, transfection, immunostaining, Luciferase assay and RNAi, FRET assay, IP and western blot analysis

S2 cells were cultured at 25 °C in a Schneider’s Drosophila Medium (Invitrogen) with 10% fetal bovine serum, 100 U/ml penicillin, 100 μg/ml streptomycin. Ptc-related plasmids transfection and dsRNA transfection were performed using the lipo3000 according to manufacturer’s instructions.

For S2 cell immunostaining analysis, cells were fixed in 4% formaldehyde for 10 min and permeabilized in 0.25% Triton X-100 for 2 min (if cell-surface immunostaining, ignore the permeabilization step). The cells were blocked in 1% BSA in PBS for 30 min and incubated in primary and secondary antibody for 2 and 1 h, respectively. Images were captured by confocal microscopy.

For the dual luciferase reporter assay, 1 ml S2 cells in 24 well (1.0 × 10^6^) were transfected with 0.3 μg ub-Gal4, 0.05 μg Ci, 0.025 μg Sufu, 0.05 μg Ptc reporter, 0.005 μg Renilla, 1 μg Ptc-related plasmid, 2 μg dsRNA of ptc 5′UTR, then cells were lysed in indicated buffer and next the luciferase assays were performed as standard procedures.

For the FRET assay, S2 cells were transfected with ub-Gal4, Myr-AKAR3 and related PKAc mutants. Cells were washed with PBS, fixed with 4% formaldehyde for 10 min, and mounted on the slides in 40–50% glycerol. The intensity of CFP emission before (BP) and after (AP) photobleaching of YFP with 514 nm laser at full strength for ten times was acquired through the standard procedure built in Leika TCS SP8 WLL. FRET% = [(CFP_AP_ − CFP_BP_)/CFP_AP_] × 100. Each data point was calculated using 15–20 individual cells.

For the IP and western blot assay, S2 cells were lysed in NP-40 buffer (50 mM Tris–Cl pH 8.0, 0.1 M NaCl, 10 mM NaF, 1 mM Na_3_PO4, 1% NP-40, 10% Glycerol, 1.5 mM EDTA, Protease Inhibitor) for 30 min at 4 °C. After centrifugation, the supernatants were incubated with indicated primary antibodies for 2 h and protein A/G gel for 1 h at 4 °C. After affinity pull-down, gels were washed in lysis buffer for 10 min three times and then boiled in 40 μl 1× SDS loading buffer. Western blot analysis was carried out by SDS-PADE electrophoresis by using standard protocol.

### Statistical analysis

Data were presented as Mean ± SD and statistical significance was calculated using two-tailed Student’s *t* test. *P*-values are presented in figures, *P < 0.05, **P < 0.01, *N.S*., not significant.

## Supplementary information

Supplementary figures and data.

## Supplementary information

**Supplementary Information** accompanies the paper at (10.1038/s41421-019-0112-z).
