# Supplementary material for: PKAc-directed interaction and phosphorylation of Ptc is required for Hh signaling inhibition in Drosophila
Source: Cell Discov. 2019 Sep 10;5:44. doi: 10.1038/s41421-019-0112-z (PMC6796939; doi:10.1038/s41421-019-0112-z)
Supplement: Supplementary file 1 — Supplementary figures and data. [file 41421_2019_112_MOESM1_ESM.pdf]

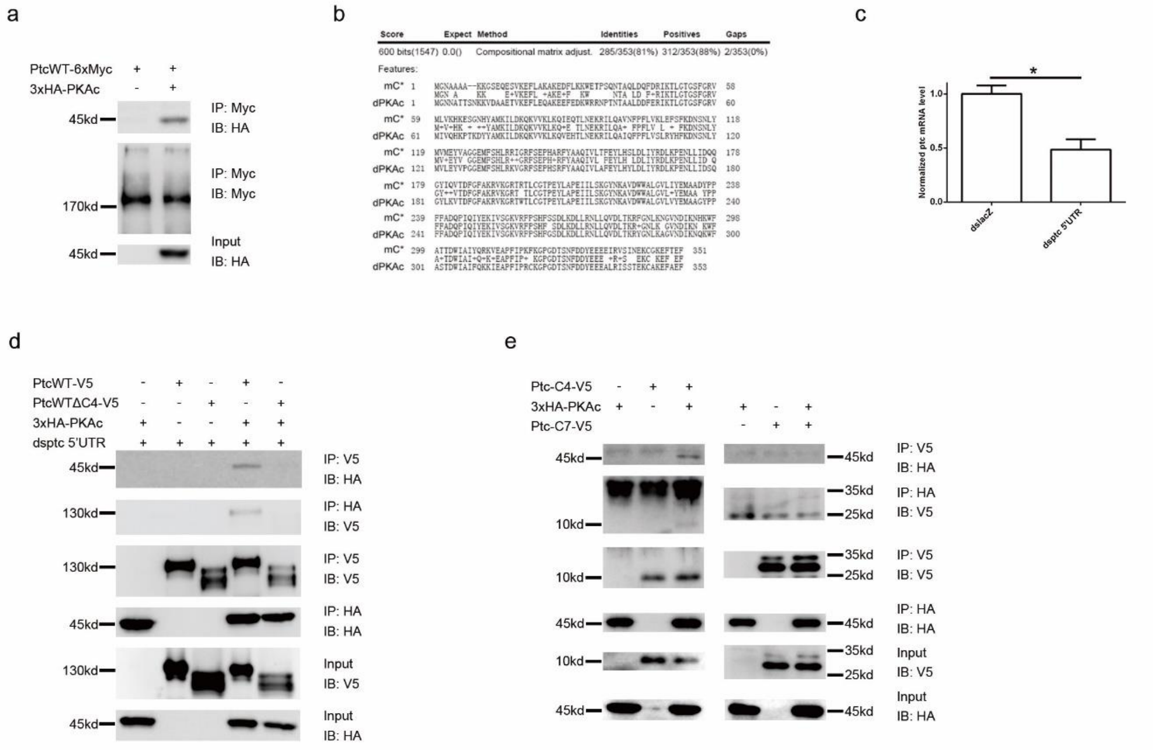

Supplementary Fig S1. (supplementary to Fig 1)Physical interaction between Ptc and PKAc.

(A) Immunoprecipitation assays to detect interaction between 3×HA-PKAc and PtcWT-6×Myc by western blot.

(B) The amino acid sequence alignment identified over 80% sequence identity between *Drosophila*PKAc and mammalian active form of PKAc (mC*).

(C) RT-qPCR analysis of RNAi efficiency of dsRNA against ptc 5’UTR in S2 cells.

(D) S2 cells were transfected with dsRNA against ptc 5’UTR to knockdown endogenous Ptc and co-transfected with indicated expression constructs including 3×HA-PKAc, PtcWT-V5 and PtcWTΔC4-V5. Immunoprecipitation assays to detect interaction between 3×HA-PKAc and PtcWT-V5 or PtcWTΔC4-V5 by western blot.

(E) S2 cells were transfected with the indicated constructs including 3×HA-PKAc, Ptc-C4-V5 and Ptc-C7-V5, followed by co-Immunoprecipitation with anti-V5/HA antibodies. Western blot to detect interactions between PKAc and Ptc-C4 or Ptc-C7.

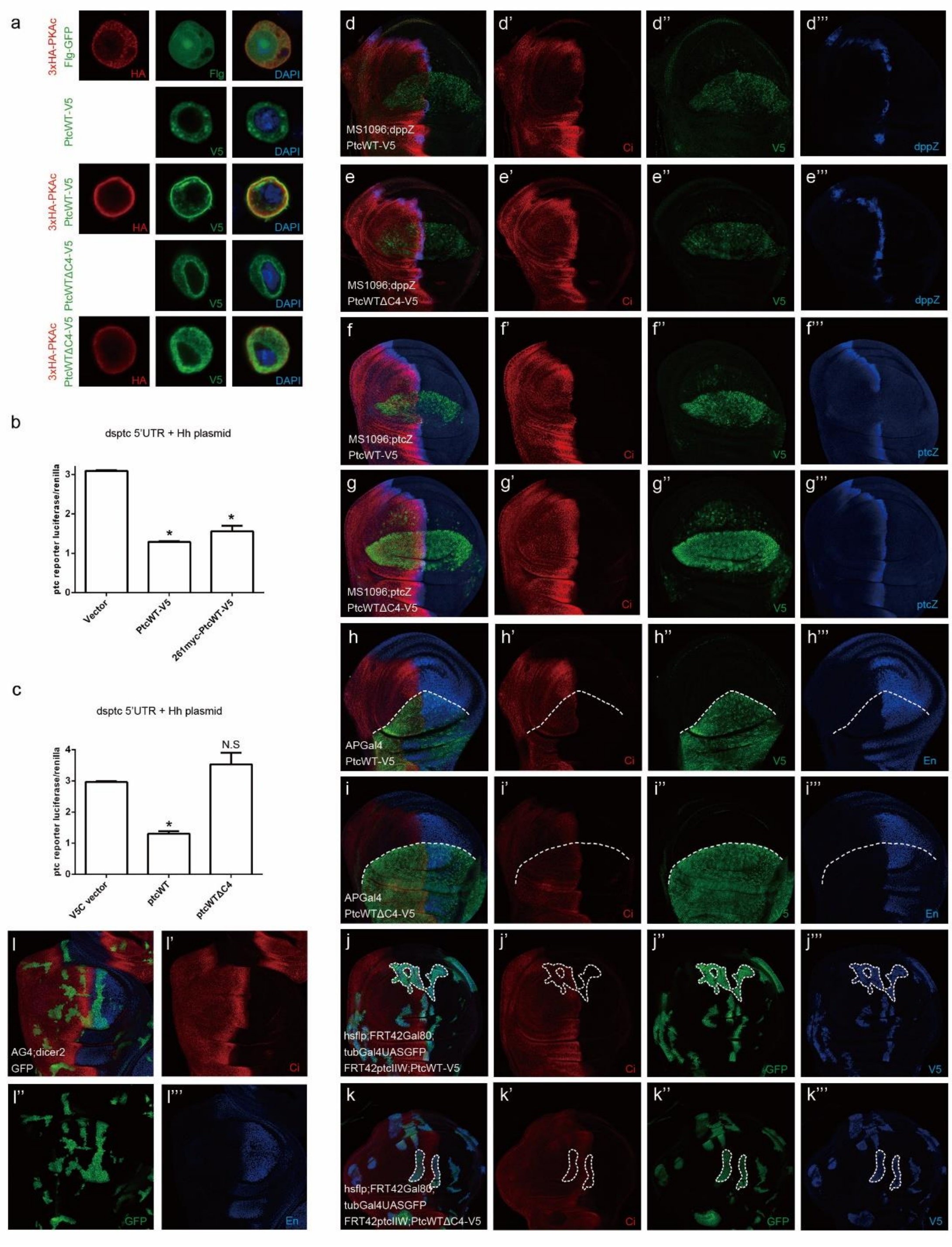

Supplementary Fig S2. (supplementary to Fig 2)Intracellular C4 domain of Ptc is required for Ptc inhibitory function on Hh signaling.

(A) Immunostaining of 3×HA-PKAc and PtcWT-V5 or PtcWTΔC4-V5 to detect the

influence of Ptc-PKAcinteraction on Ptc membrane location.

(B) The dual-luciferase detection of Ptc reporter when overexpressing PtcWT-V5, Myc-PtcWT-V5, Hh plasmid, dsRNA against ptc 5’UTR. *, p value<0.05 versus Vector.

(C) The dual-luciferase detection of Ptc reporter when overexpressing PtcWT, PtcWTΔC4, Hh plasmid, dsRNA against ptc5’UTR. *, p value<0.05, N.S., p value>0.05 versus V5C vector respectively.

(D-E’’’) Wing discs expressing PtcWT-V5 (D-D’’’) or PtcWTΔC4-V5 (E-E’’’) driven by the MS1096; dpp-lacZ were immunostained to show Ci and dpp-lacZ (dppZ) levels.

(F-G’’’) Wing discs expressing PtcWT-V5 (F-F’’’) or PtcWTΔC4-V5 (G-G’’’) driven by the MS1096; ptc-lacZ were immunostained to show Ci and ptc-lacZ (ptcZ) levels.

(H-I’’’) Wing discs expressing PtcWT-V5 (H-H’’’) or PtcWTΔC4-V5 (I-I’’’) driven by the dorsal compartment-specific driver AP-Gal4 were immunostained to show Ci and En levels.

(J-K’’’) Rescue assays to detect the rescue ability of PtcWT-V5 or PtcWTΔC4-V5 to loss-of-inhibition induced by endogenous Ptc depletion. Ci levels were detected in GFP clones with PtcWT overexpression plus Ptc depletion (J-J’’’) or PtcWTΔC4 overexpression plus Ptc depletion (K-K’’’).

(L-L’’’) Wing discs expressing GFP driven by the AG4; dicer2 were immunostained to show Ci and En levels as the negative control for Fig 2I, 2J, 4I, 4J.

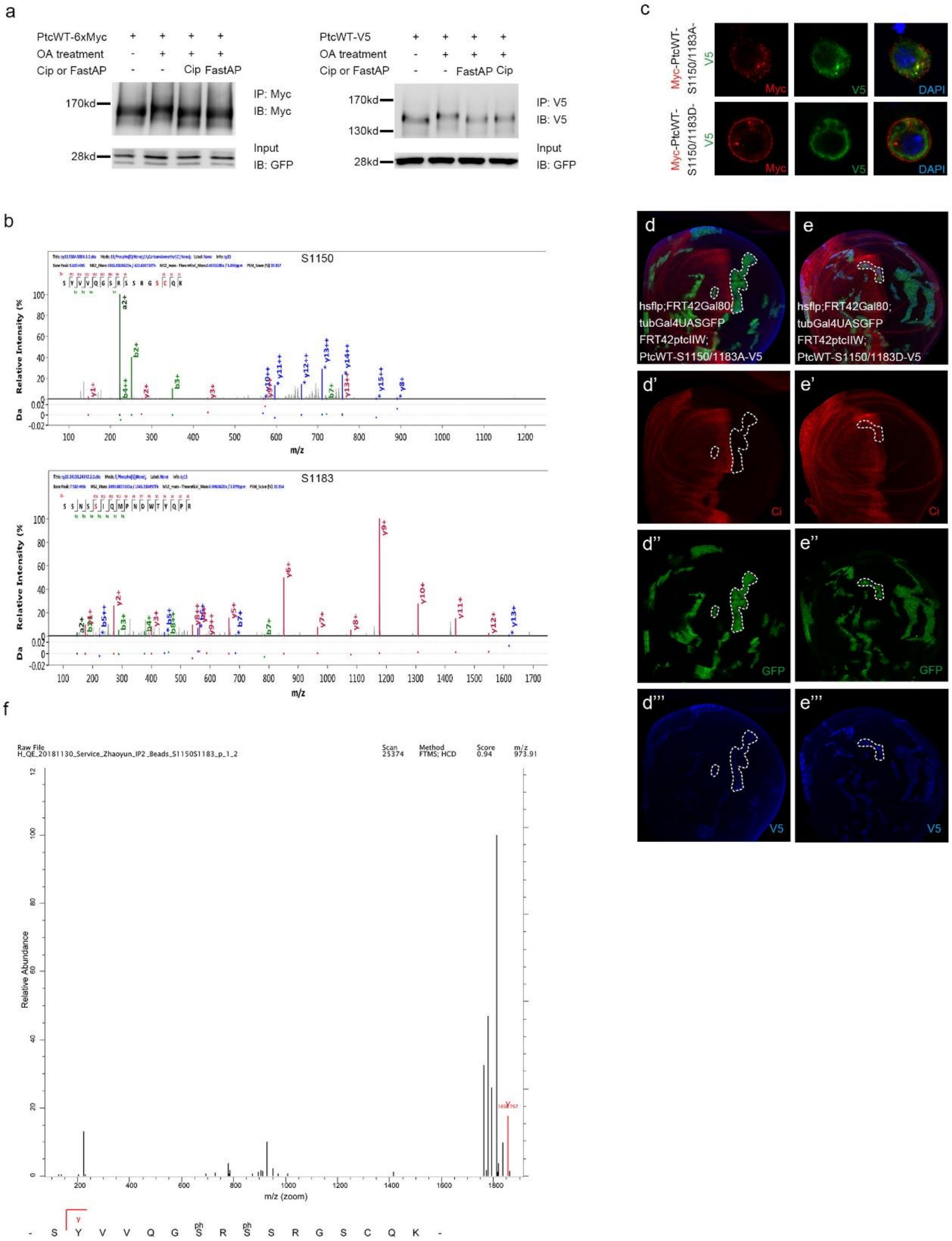

Supplementary Fig S3. (supplementary to Fig 3) Phosphorylation on Ser1150/1183 is required for Ptc inhibitory function on Hh signaling.

(A) Phosphorylation assays for overexpressed PtcWT-6×Myc (left)or PtcWT-V5 (right) in

S2 cells. The cells were treated with OA(Okadaic Acid, Sigma) to induce phosphorylation or with Cip or FastAP to induce dephosphorylation.

(B) The mass spectrometry results of the phosphorylation modification on S1150 and S1183 in the purified GST-ptc-C7 sample (B).

(C) Cell Surface Immunostaining of Myc-PtcWT-S1150/1183A-V5 and Myc-PtcWT-S1150/1183D-V5 in S2 cells.

(D-D’’’) Rescue assays to detect the rescue ability of PtcWT-S1150/1183A-V5 to loss-of-inhibition induced by endogenous Ptc depletion. Ci levels were detected in GFP clones with PtcWT-S1150/1183A-V5 overexpression plus Ptc depletion.

(E-E’’’) Rescue assays to detect the rescue ability of PtcWT-S1150/1183D-V5 to loss-of-inhibition induced by endogenous Ptc depletion. Ci levels were detected in GFP clones with PtcWT-S1150/1183D-V5 overexpression plus Ptc depletion.

(F) The mass spectrometry results of the phosphorylation modification on S1150 and S1183 in the overexpressed PtcWT-6×Myc protein sample in S2 cells.

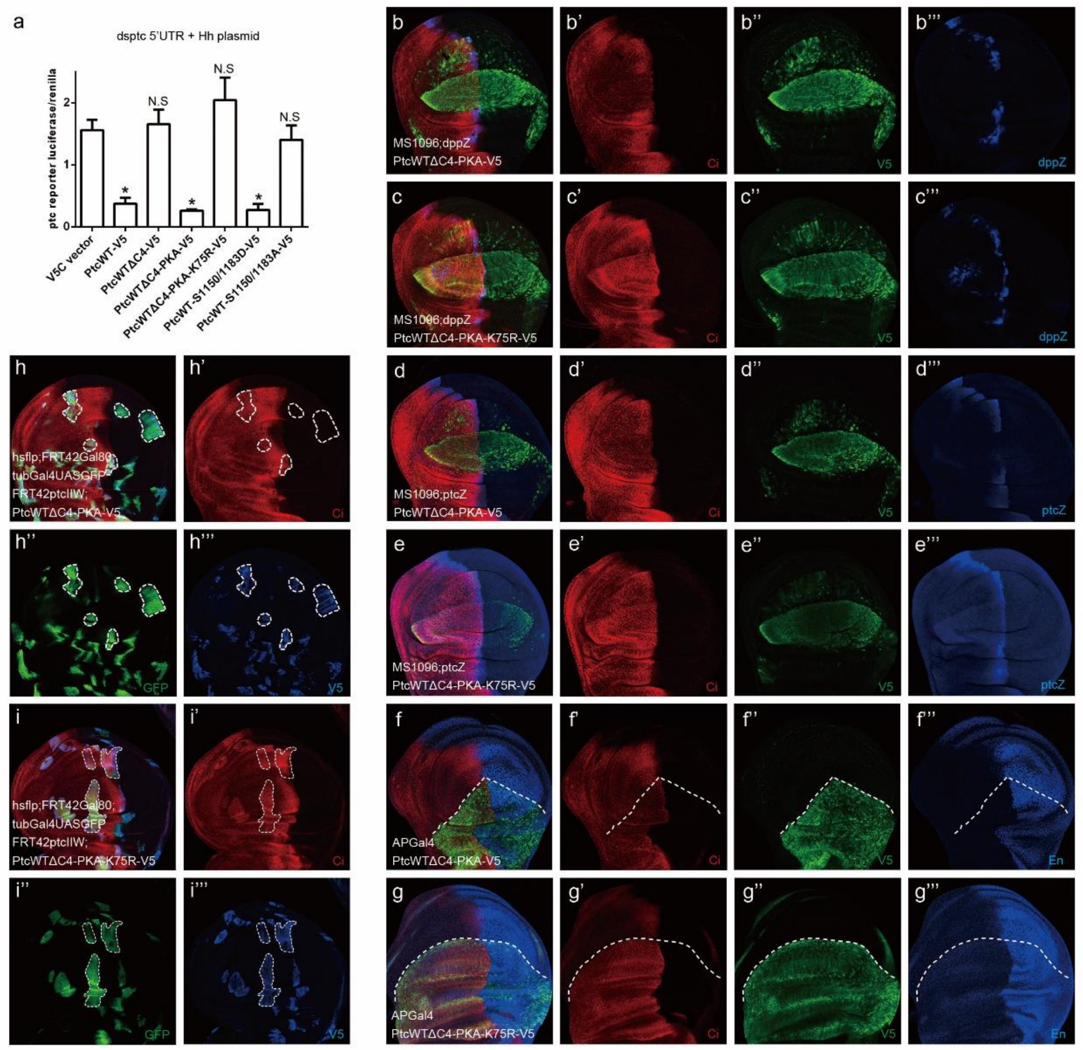

Supplementary Fig S4. (supplementary to Fig 4) PKAc kinase activity is required for Ptc inhibitory function on Hh signaling.

(A) The dual-luciferase detection of Ptc reporter when overexpressing PtcWT, PtcWTΔC4, PtcWTΔC4-PKA, PtcWTΔC4-PKA-K75R,PtcWT-S1150/1183A, PtcWT-S1150/1183D, Hh plasmid, dsRNA against ptc 5’UTR.*, p value<0.05, N.S., p value>0.05 versus V5C vector respectively.

(B-C’’’) Wing discs expressing PtcWTΔC4-PKA-V5 (B-B’’’) or PtcWTΔC4-PKA-K75R-V5 (C-C’’’) driven by the MS1096; dpp-lacZ were immunostained to show Ci and dpp-lacZ (dppZ).

(D-E’’’) Wing discs expressing PtcWTΔC4-PKA-V5 (D-D’’’) or PtcWTΔC4-PKA-K75R-V5 (E-E’’’) driven by the MS1096; ptc-lacZ were immunostained to show Ci and ptc-lacZ (ptcZ).

(F-G’’’) Wing discs expressing PtcWTΔC4-PKA-V5 (F-F’’’) or PtcWTΔC4-PKA-K75R-V5 (G-G’’’) driven by the dorsal compartment-specific driver AP-Gal4 were immunostained to show Ci and En.

(H-I’’’) Rescue assays to detect the rescue ability of PtcWTΔC4-PKA-V5 or PtcWTΔC4-PKA-K75R-V5 to loss-of-inhibition induced by endogenous Ptc depletion. Ci levels were detected in GFP clones with PtcWTΔC4-PKA-V5 overexpression plus Ptc depletion (H-H’’’) or with PtcWTΔC4-PKA-K75R-V5 overexpression plus Ptc depletion (I-I’’’).

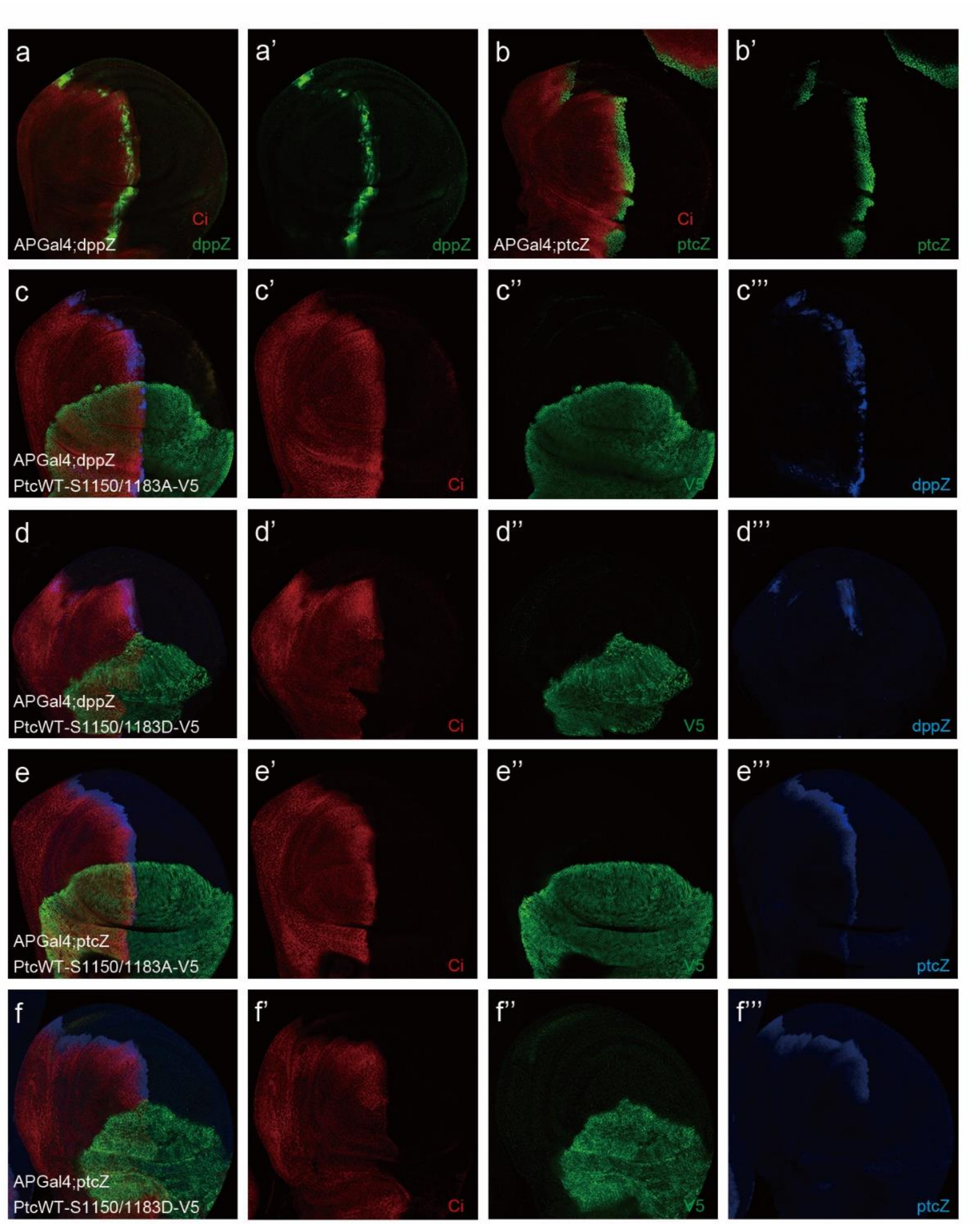

Supplementary Fig S5. (supplementary to Fig 4) The function of Ptc phosphorylation site mutations on Hh signaling

(A-B’) Wing discs immunostaining of APGal4; dpp-lacZ (A-A’) or APGal4; ptc-lacZ (B-B’) to show Ci (red) and lacZ (green) levels as the negative controls for Fig S5C-F’’’.

(C-D’’’) Immunostaining of wing discs expressing PtcWT-S1150/1183A-V5(C-C’’’, green) or PtcWT-S1150/1183D-V5 (D-D’’’, green) driven by the dorsal compartment-specific driver AP-Gal4; Ci (red) and dpp-lacZ (blue) were immunostainedto show the inhibitory effects of PtcWT-S1150/1183A-V5 or PtcWT-S1150/1183D-V5 on Hh signaling *in vivo*.

(E-F’’’) Immunostaining of wing discs expressing PtcWT-S1150/1183A-V5(E-E’’’, green) or PtcWT-S1150/1183D-V5 (F-F’’’, green) driven by the dorsal compartment-specific driver AP-Gal4; Ci (red) and ptc-lacZ (blue) were immunostainedto show the inhibitory effects of PtcWT-S1150/1183A-V5 or PtcWT-S1150/1183D-V5 on Hh signaling *in vivo*.

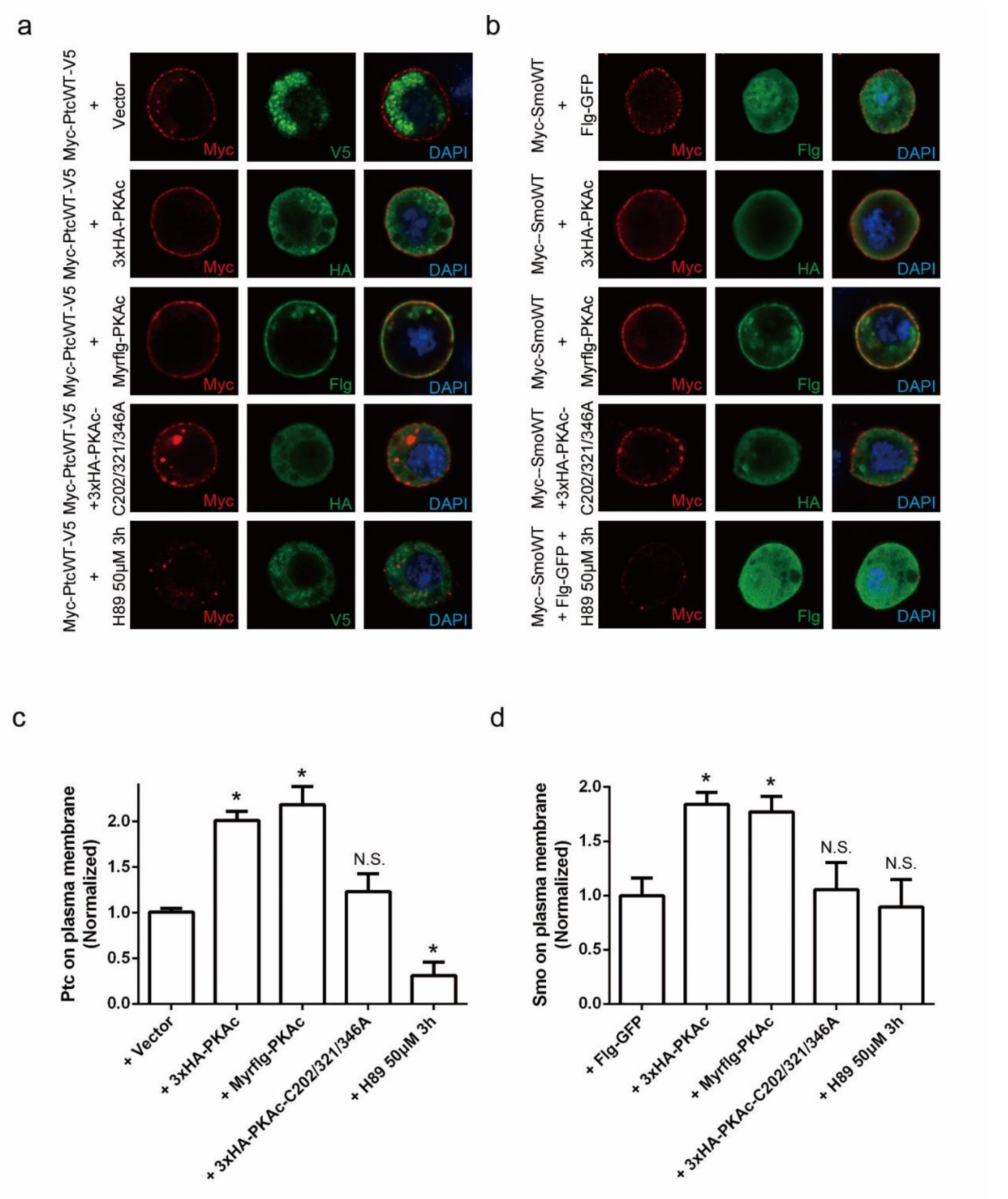

Supplementary Fig S6. (supplementary to Fig 5) The effects of PKAc on plasma membrane accumulation of Ptc and Smo

(A, C) Cell surface immunostaining of Myc-PtcWT-V5 when respectively cotransfected with vector, 3×HA-PKAc, Myrflg-PKAc, 3×HA-PKAc-C202/321/346A or treated with PKAcinhibitor H89. The normalized data statistics of Ptc immunostaining on plasma

membrane, *, p value<0.05, N.S., p value>0.05 versus Vector respectively (C).

(B, D) Cell surface immunostaining of Myc-Smo when respectively cotransfected with Flag-GFP, 3×HA-PKAc, Myrflg-PKAc, 3×HA-PKAc-C202/321/346A or treated with PKAc inhibitor H89. The normalized data statistics of Smo immunostaining on plasma membrane, *, p value<0.05, N.S., p value>0.05 versus Vector respectively (D).

Materials and Methods

Fly mutants and stocks

All fly stocks were raised on standard yeast-based food at 25°C.*MS1096-, act>CD2>Gal4 (AG4), ap-Gal4* drivers have been described[1,2,3,4]. *UAS-Flag-SmoWT, UAS-Flag-SmoSD, UAS-Myrflg-mC*, UAS-Flag-PKaC1*(Bloomington, #35554)*, dpp-lacZ, ptc-lacZ* have been described[2,3,4,5,6,7,8].

Mutant clones were generated by standard FLP (flippase)/FRT (flippase recognition target)-mediated mitotic recombination as previously described[9] using the next genotypes: hsflp; FRT42Gal80/FRT42ptcIIW; tubGal4UASGFP/TM6B (or PtcWT-V5, PtcWTΔC4-V5, PtcWTΔC4-PKA-V5, PtcWTΔC4-PKA-K75R-V5).

Constructs for cell transfection and fly transgenes

The vectors of pUAST-3xFlg, pUAST-3xHA, pUAST-6xMyc, pUAST-V5c were used to generate constructs for overexpression of HA-dPKAc, Myc-dPKAc, Myc-dPKAc-C202A, Myc-dPKAc-C321A, Myc-dPKAc-C346A, PtcWT-V5, 261Myc-PtcWT-V5, PtcWT-6xMyc, PtcWTΔC1-V5, PtcWTΔC2-V5, PtcWTΔC3-V5, PtcWTΔC4-V5, PtcWTΔC6-V5, Ptc1196aaΔC4-V5, Ptc-C4-V5, Ptc-C7-V5, Myrflg-Ptc-C4, Myrflg-Ptc-C7, PtcWTΔC4-PKA-V5, PtcWTΔC4-PKA-K75R-V5, Myr-Ptc-C4-V5.

The vectors of pGEX-4T-1 and pET28a(+) were used to generate constructs for protein expression of GST-Ptc-C1, GST-Ptc-C4, GST-Ptc-C7, His-dPKAc, His-Sumo-Ptc-C4, His-Sumo-dPKAc.

The primers used for dsRNA of ptc 5’UTR are as follows:

dslacZ-CDS-5 TAATACGACTCACTATAGGgATGAGCGTGGTGGTTATGCCGATC
dslacZ-CDS-3 TAATACGACTCACTATAGGgCCATGCCGTGGGTTTCAATATTGG
dsptc-5’UTR-5 TAATACGACTCACTATAGGgCACGGATGCGAGGCGAAGTTC
dsptc-5’UTR-3 TAATACGACTCACTATAGGgCTTGGGAGACAGTGTGCGGATTG

The *attB* vector was used to generate all the ptc related transgenic constructs, attb-PtcWT-V5, attb-PtcWTΔC4-V5, attb-PtcWTΔC4-PKA-V5, attb-PtcWTΔC4-PKA-K75R-V5. All these plasmids were prepared for generating flies with transgenes inserted at the 25C and 86F *attP*locus[10], The Myr-Ptc-C4-V5, Myc-dPKAc-C202/321/346A and Myrflg-dPKAc-C202/321/346A transgenic fly was generated using pUAST vector by standard P-element mediated transformation[11].

S-Palmitoylation assay

The S-palmitoylation assay was performed according to the protocols described previously[12,13]. S2 cells transfected with Myc-tagged proteins were collected by centrifugation at 500g for 5 min, and the pellet was homogenized in lysis buffer [10 mM sodium phosphate, 2 mM Na_2EDTA, 0.32 M sucrose, 1% Triton X-100, and protease inhibitors cocktails from Roche]. Next, 50 mM N-ethylmaleimide was added for blocking free sulfhydryl groups, and then were incubated using anti-Myc beads at 4°C for 4 h, the beads were washed three times with cold lysis buffer and then incubated with elution buffer (1% SDS, 10 mM sodium phosphate, 2 mM Na_2EDTA, 0.32 M sucrose) at 50°C for 5 min to elute Myc-tagged proteins. Eluted samples were divided into two equal portions: one treated with 1 M hydroxylamine (pH 7.4) and the other with 1 M Tris·HCl (pH 7.4) both with the presence of activated thiol-Sepharose 6B (Sigma). After a 2-h incubation at room temperature, Beads were then washed three times with washing buffer (10 mM sodium phosphate, 2 mM Na2EDTA, 0.32 M sucrose, 1% Triton X-100, 500 mM NaCl). Samples were analyzed by Western blots, and the Myc antibody was used to detect the presence of Myc-tagged proteins.

Biotin Switch Assay

The biotin switch assay was described previously[12]. S2 cells transfected with Myc-tagged proteins were collected by centrifugation at 500g for 5 min, and the cell pellet was homogenized in lysis buffer [10 mM sodium phosphate, 2 mM Na_2EDTA, 0.32 M sucrose, 1% Triton X-100, 50mM N-ethylmaleimide and protease inhibitors cocktails

from Roche] and incubated at 4°C for 6 h to pre-block free sulfhydryl groups. Then the Lysates were sonicated for 30 s on ice followed by centrifugation at 12,000rpm for 10 min. Next, the 15µl of Anti-Myc beads incubated with the lysates supernatant overnight, rotating at 4 °C. The beads were washed three times with cold wash buffer (10 mM sodium phosphate, 2 mM Na_2EDTA, and protease inhibitors, pH 7.4) and then incubated with hydrolysis-labeling buffer (1 M hydroxylamine, 80 µM Biotin-BMCC, 10 mM sodium phosphate, 2 mM Na_2EDTA, and protease inhibitors, pH 7.4, As a control, 1 M Tris-HCl was substituted for hydroxylamine) at room temperature for 2 h. Beads were then washed three times with wash buffer and incubated with SDS-PAGE loading buffer at 70°C for 15 min to elute proteins. Samples were analyzed by SDS-PAGE and Western blotting. A biotin antibody was used to detect S-palmitoylation.

GST fusion protein pull-down assay

GST fusion proteins and 6xHis fusion proteins were expressed in *E. coli BL21* and purified with glutathione agarose beads (GE) and Ni Sepharose (GE), respectively. The GST fusion protein loaded beads were incubated with the same protein level of purified His-PKAc in GST pull down buffer (50mM Tris-Cl pH 8.0, 0.1M NaCl, 10mM NaF, 1mM Na_3PO4, 1% NP-40, 10% Glycerol, 1.5mM EDTA) at $4^{O}C$ for 2h. The beads were washed 10min x 3 times with buffer above. Western blot analysis was then performed to detect the direct interaction between PKAc and Ptc-C1, Ptc-C4, Ptc-C7.

***In vitro* phosphorylation kinase assay using ATP analog ATPγS**

Prepared the purified substrates and reaction mixture as follows: 10x PKA Reaction Buffer (New England Biolabs), PKA catalytic subunit (New England Biolabs), $MgCl_2$ (10mM final),ATPγS (1mM final, ab138911, Abcam), Purified substrates 0.5ug, and incubated for 45min at $30^{o}C$.Added 1ul 50mM PNBM (ab138910, Abcam), and incubated for 1h at room temperature.Added 7ul 4x SDS loading buffer and boiled the sample. 10ul sample was loaded to SDS PAGE and western blot was performed. Detected thiophosphorylation by anti-thiophosphorylation ester rabbit monoclonal antibody

1:5000 (ab92570, Abcam).

Mass spectrometry analysis of phosphorylation sites

Protein samples were lysed by mixing with 50 μL SDT lysis solution followed by incubation at 95°C for 3 min. The DNA was sheared by sonication to reduce the viscosity of the sample. Before sample processing, the lysate was clarified by centrifugation at 16,000g for 5 min. Then, protein samples were digested by sequencing grade modified trypsin (1:50) using the Filter-Aided Sample Preparation protocol[14]. Peptide samples were cleaned of salts using stop and go extraction tips, dried, and then stored at −80°C before analysis[15].

Peptide samples were analyzed on Q Exactive mass spectrometer (Thermo Fisher Scientific, USA). Peptide mixtures were separated through a nano-emitter column (15 cm length, 75 μM inner diameter) packed in-house with 3 μM C18 ReproSil particles and introduced into the mass spectrometer using a nanoelectrospray ion source. A linear gradient from 2 to 35% buffer B (buffer A, 0.1% formic acid in ddH_2O; buffer B, 0.1% formic acid in acetonitrile) was used for peptide separation at a flow rate of 300 nL/min. HCD-based fragmentation was performed, which was set to alternate between a full scan followed by up to twenty fragmentation scans. Dynamic exclusion was enabled to void choosing former target ions, and lock-mass was enabled using 445.120025. Raw MS data were processed with MaxQuantsoftware[16] version 1.6.2.10 using the default settings with minor changes: phosphorylation (STY), oxidation (M) and acetylation (protein N-term) were selected as variable modifications, and carbamidomethyl (C) was selected as fixed modification. Database searching was performed using the Andromeda search engine in MaxQuant software against fasta file of the targeted protein (P18502) from Swiss-Prot Homo sapiens sequence database. MS and MS/MS figures were visualized in MaxQuant software and manual checked.

Reference

1. Calleja M, Moreno E, Pelaz S, Morata G. Visualization of gene expression in living adult Drosophila. Science. 1996; 274:252–255.

2. Zhang Z, Lv X, Yin WC, Zhang X, Feng J, Wu W, Hui CC, Zhang L, Zhao Y. Ter94 ATPase complex targets k11-linked ubiquitinated ci to proteasomes for partial degradation. Developmental cell. 2013;25:636-644.

3. Zhang Z, Feng J, Pan C, Lv X, Wu W, Zhou Z, Liu F, Zhang L, Zhao Y. Atrophin-Rpd3 complex represses Hedgehog signaling by acting as a corepressor of CiR. The Journal of Cell Biology.2013;203:575-583.

4. Yang X, Mao F, Lv X, Zhang Z, Fu L, Wu W, Zhou Z, Zhang L, Zhao Y. Drosophila Vps36 regulates Smo trafficking in Hedgehog signaling. Journal of Cell Science.2013;126:4230-4238.

5. Jia J, Tong C, Wang B, Luo L, Jiang J. Hedgehog Signaling Activity of Smoothened Requires Phosphorylation by Protein Kinase A and Casein Kinase I. Nature. 2004; 432:1045–1050.

6. Cross FR, Garber EA, Pellman D, Hanafusa H. A short sequence in the p60src N terminus is required for p60src myristylation and membrane association and for cell transformation. Molecular and Cellular Biology. 1984; 4:1834–1842.

7. Simon MA, Drees B, Kornberg T, Bishop JM. The nucleotide sequence and the tissue-specific expression of Drosophila c-src. Cell. 1985; 42:831–840.

8. Li S, Ma G, Wang B, Jiang J. Hedgehog induces formation of PKA-Smoothened complexes to promote Smoothened phosphorylation and pathway activation. ScienceSsignaling.2014;7, ra62.

9. Theodosiou NA, Xu T. Use of FLP/FRT system to study Drosophila development. Methods. 1998;14(4):355-65.

10. Bischof J, Maeda RK, Hediger M, Karch F, Basler K. An optimized transgenesis system for Drosophila using germ-line-specific phiC31 integrases. Proc Natl Acad Sci U S A. 2007;104:3312–3317.

11. Rubin GM, Spradling AC. Genetic transformation of Drosophila with transposable element vectors. Science. 1982; 218:348–353.

12. He M, Jenkins P, Bennett V. Cysteine 70 of ankyrin-G is S-palmitoylated and is required for function of ankyrin-G in membrane domain assembly. J Biol Chem 2012;287: 43995–44005

13. Li W, Li W, Zou L, Jia S, Li C, Liu K, Zhang G, Sun Q, Xiao F, Chen D. Membrane targeting of inhibitory Smads through palmitoylation controls TGF-β/BMP signaling. Proc Natl Acad Sci U S A.2017; 114(50):13206-13211.

14. Wiśniewski JR, Zougman A, Nagaraj N, Mann M. Universal sample preparation method for proteome analysis. Nature Methods. 2009; 6:359-362.

15. Rappsilber J, Ishihama Y, Mann M. Stop and go extraction tips for matrix-assisted laser desorption/ionization, nanoelectrospray, and LC/MS sample pretreatment in proteomics. Analytical Chemistry. 2003; 75: 663-670.

16. Cox J, Matic I, Hilger M, Nagaraj N, Selbach M, Olsen JV, Mann M. A practical guide to the MaxQuant computational platform for SILAC-based quantitative proteomics. Nature Protocols. 2009; 4, 698-705.
